# Transcriptomic analysis of *short*-*fruit 1* (*sf1*) reveals new insights into the variation of fruit-related traits in *Cucumis sativus*

**DOI:** 10.1038/s41598-017-02932-5

**Published:** 2017-06-07

**Authors:** Lina Wang, Chenxing Cao, Shuangshuang Zheng, Haiyang Zhang, Panjing Liu, Qian Ge, Jinrui Li, Zhonghai Ren

**Affiliations:** State Key Laboratory of Crop Biology; Key Laboratory of Biology and Genetic Improvement of Horticultural Crops (Huanghuai Region), Ministry of Agriculture; College of Horticulture Science and Engineering, Shandong Agricultural University, No.61 Daizong Road, Tai’an, Shandong 271018 China

## Abstract

Fruit size is an important quality trait in different market classes of *Cucumis sativus* L., an economically important vegetable cultivated worldwide, but the genetic and molecular mechanisms that control fruit size are largely unknown. In this study, we isolated a natural cucumber mutant, *short fruit 1* (*sf1*), caused by a single recessive Mendelian factor, from the North China-type inbred line CNS2. In addition to significantly decreased fruit length, other fruit-related phenotypic variations were also observed in *sf1* compared to the wild-type (WT) phenotype, indicating that *sf1* might have pleiotropic effects. Microscopic imaging showed that fruit cell size in *sf1* was much larger than that in WT, suggesting that the short fruit phenotype in *sf1* is caused by decreased cell number. Fine mapping revealed that *sf1* was localized to a 174.3 kb region on chromosome 6. Similarly, SNP association analysis of bulked segregant RNA-Seq data showed increased SNP frequency in the same region of chromosome 6. In addition, transcriptomic analysis revealed that *sf1* might control fruit length through the fine-tuning of cytokinin and auxin signalling, gibberellin biosynthesis and signal transduction in cucumber fruits. Overall, our results provide important information for further study of fruit length and other fruit-related features in cucumber.

## Introduction

Cucumber (*Cucumis sativus* L., 2n = 14), a member of the family *Cucurbitaceae*, is one of the most economically important vegetable crops cultivated throughout the world. As one of the most significant fruit-related traits, fruit length is a key consideration in cucumber breeding. Previous studies have demonstrated that fruit length is quantitatively inherited, and several quantitative trait loci (QTLs) have been identified and analysed. Wenzel *et al*. were the first to investigate the QTLs that affect fruit quality traits in F2:3 families from a cross between the inbred lines Gyl4 and PI432860; in their studies, three QTLs were identified and shown to be responsible for cucumber fruit length^1^. Subsequently, two QTLs associated with fruit length were identified by Serquen *et al*. in F2:3 populations developed from G421 × H-19 cucumber inbred lines^2^. By analysing recombinant inbred lines (RILs) from the same cross between G421 and H-19, Fazio *et al*. identified twelve QTLs that contribute to cucumber fruit length^3^. Yuan *et al*.^4^ constructed F2 and F2:3 families from a narrow cross between inbred lines S94 and S06 and identified one QTL for fruit length in the F2 population and six QTLs in F2:3 families, three of which affected fruit length in autumn and three of which affected fruit length in spring. Additionally, five QTLs were identified and shown to be responsible for mature fruit length in four environments using an RIL population developed from CC3 × SWCC8 cucumber inbred lines^5^. With the aid of QTL models, 12 consensus QTLs were also shown to determine cucumber fruit length^6^. In addition, transcriptomic analysis revealed that cucumber fruit length might be regulated by microtubules, CDKs-cyclins, and expansin-mediated cell division and cell expansion and that some transcription factors might function as key upstream players^7^.

Fruit growth and development are closely associated with cell division and expansion, and cell division and expansion can be modulated by plant hormones such as cytokinins, gibberellins and auxin. For example, it has been reported that cytokinin concentration and the expression of genes associated with cytokinin biosynthesis are maintained at high levels during early tomato fruit development, and different temporal patterns of expression of different classes of cytokinins and genes associated with their biosynthetic pathways have been observed, providing evidence for a significant role of cytokinin signalling in the cell division phase of tomato fruit development^8^. A positive relationship between endogenous cytokinin level and cucumber fruit size has also been demonstrated^9^. Gibberellins control both cell proliferation and expansion rates, processes that rely on the destruction of DELLAs^10^. Significant roles for gibberellin biosynthesis and signalling in both cell division and cell expansion have also been observed during fruit growth and development^11, 12^. In general, the results of studies showing that auxin functions significantly in plant cell division and expansion are well accepted^13^, whereas evidence that the coordination of plant hormones is involved in the regulation of fruit length is still ambiguous.

In this study, we obtained a spontaneous mutant, *short fruit 1* (*sf1*), from a cucumber inbred line CNS2 with long fruits (wild type, WT) and characterized the *sf1* mutant through comprehensive methodologies, including genetics, physiology, histology and comparative transcriptomics. Based on the results, we proposed a new mechanism by which *sf1* might control fruit length in cucumber by fine-tuning cytokinin and auxin signalling and by regulating gibberellin biosynthesis and signal transduction.

## Results

### Isolation and phenotypic analysis of the *sf1* mutant

**Figure 1:**
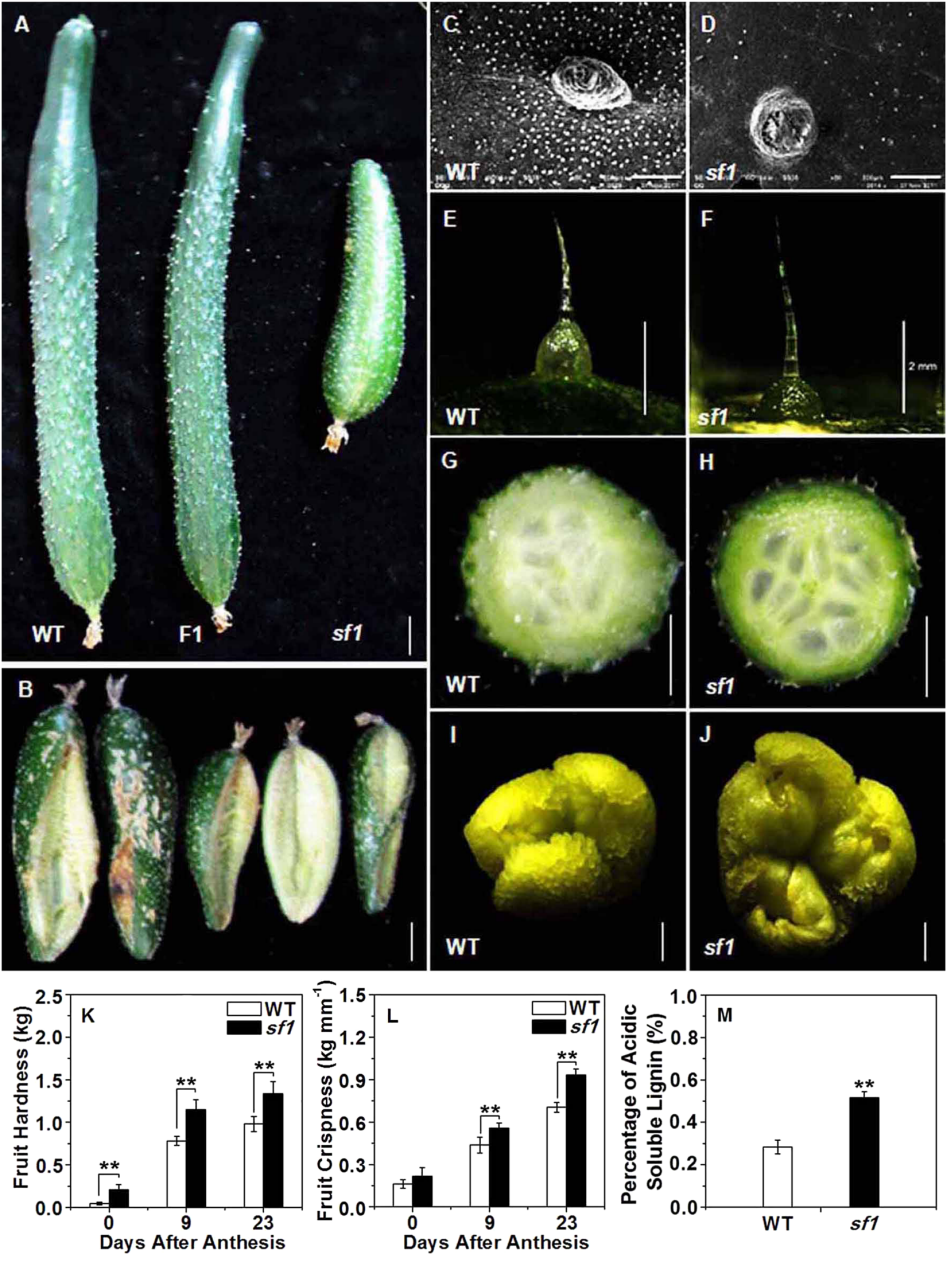
Phenotypes of WT and *sf1*. (**A**) Fruit of WT, F1 and *sf1* at 11 days after anthesis (DAA). (**B**) Fruit cracking in *sf1* at 20 DAA. (**C**,**D**) Distribution of bloom trichomes on the pericarp of WT and *sf1* at 9 DAA. (**E**,**F**) Fruit spines and tubercules of WT and *sf1* at 11 DAA. (**G**,**H**) Seed cavities of WT and *sf1* fruits at 9 DAA. (**I**,**J**) Stigmas from WT and *sf1* at 0 DAA. Fruit hardness (**K**) and crispness (**L**) in WT and *sf1* at 0, 9 and 23 DAA. Acid-soluble lignin content (**M**) in WT and *sf1* cucumbers at 9 DAA. Scale bars represent 2 cm in (**A**),(**B**),(**G**) and (**H**), 2 mm in (**E**),(**F**),(**I**) and (**J**), and 500 μm in (**C**) and (**D**). ‘**’and ‘*’ indicate significant differences from WT at the 0.01 and 0.05 probability levels, respectively. Vertical bars represent standard deviation (n = 3).

We isolated a natural mutant, *sf1*, with short fruits from the cucumber inbred line CNS2, a North China type with long fruits (Fig. 1A). The fruit length of *sf1* was approximately half that of WT at the commodity maturity stage (Fig. 1A), but plant height, stem diameter, leaf area and the first female and male flower nodes of this mutant line were similar to those of the WT (Supplementary Figure 1).

In addition to decreased fruit length, many other fruit-related traits and stigma size were affected in *sf1* (Fig. 1). The fruits of *sf1* had a light green peel, no tubercle and cracked easily under moist conditions, whereas the WT fruits had a dark green peel and tubercles and did not crack at all (Fig. 1A–D,K and L). Significantly fewer bloom trichomes on the fruit surface resulted in more glossy fruits in *sf1* than in WT (Fig. 1A,C and D). There were also differences in the fruit spines of *sf1* and WT (Fig. 1E and F). A cucumber fruit spine has an upper portion with a single-cell arrangement and a warty base (Fig. 1E and F). The height of the upper portion of the *sf1* spine was 43% greater than that of WT due to an increase in the cell number in this portion of the spine, but the height of the spine base was 63% that of WT; as a result, the height of the whole spine of *sf1* was 15% greater than that of WT (Supplementary Figure 2A–D). We also found that the diameters of the stigma and seed cavity in *sf1* were 28% and 30% greater, respectively, than those in WT (Supplementary Figure 2E and G). Accordingly, the ratio of seed cavity diameter to fruit diameter increased by 53% in *sf1* relative to that in WT (Supplementary Figure 2F).

Higher chlorophyll and carotenoid content was observed in the pericarp, but more carotenoids were present in the sarcocarp of *sf1* than of WT (Supplementary Figure 2H and I). The cell wall is a protective structure of plant cells and is primarily composed of cellulose, hemicellulose and lignin^14^. Fruit hardness and crispness, two important quality parameters in cucumber, are closely related to the composition of the cell wall^15–17^. Fruit hardness refers to the capability of fruit to resist the penetration of outside objects into it. Fruit crispness refers to the capability of fruit to resist cracking when an outside force is exerted on it. In this study, *sf1* fruits displayed increased hardness and crispness (Fig. 1K and L), indicating possible involvement of *sf1* in the regulation of cell wall components. To investigate this hypothesis, we analysed the content of cell wall components in *sf1* and WT fruits at 9 days after anthesis (DAA), when obvious cracking of *sf1* fruits began to occur. Strikingly increased lignin content was observed in the *sf1* pericarp compared to that in the WT pericarp (Fig. 1M).

These significantly influenced traits of *sf1* plants were closely associated with the short-fruit phenotype, and no segregation was observed in the progenies, indicating that *sf1* might have pleiotropic effects on fruit-related traits.

### Growth and histological features of *sf1* fruit

**Figure 2:**
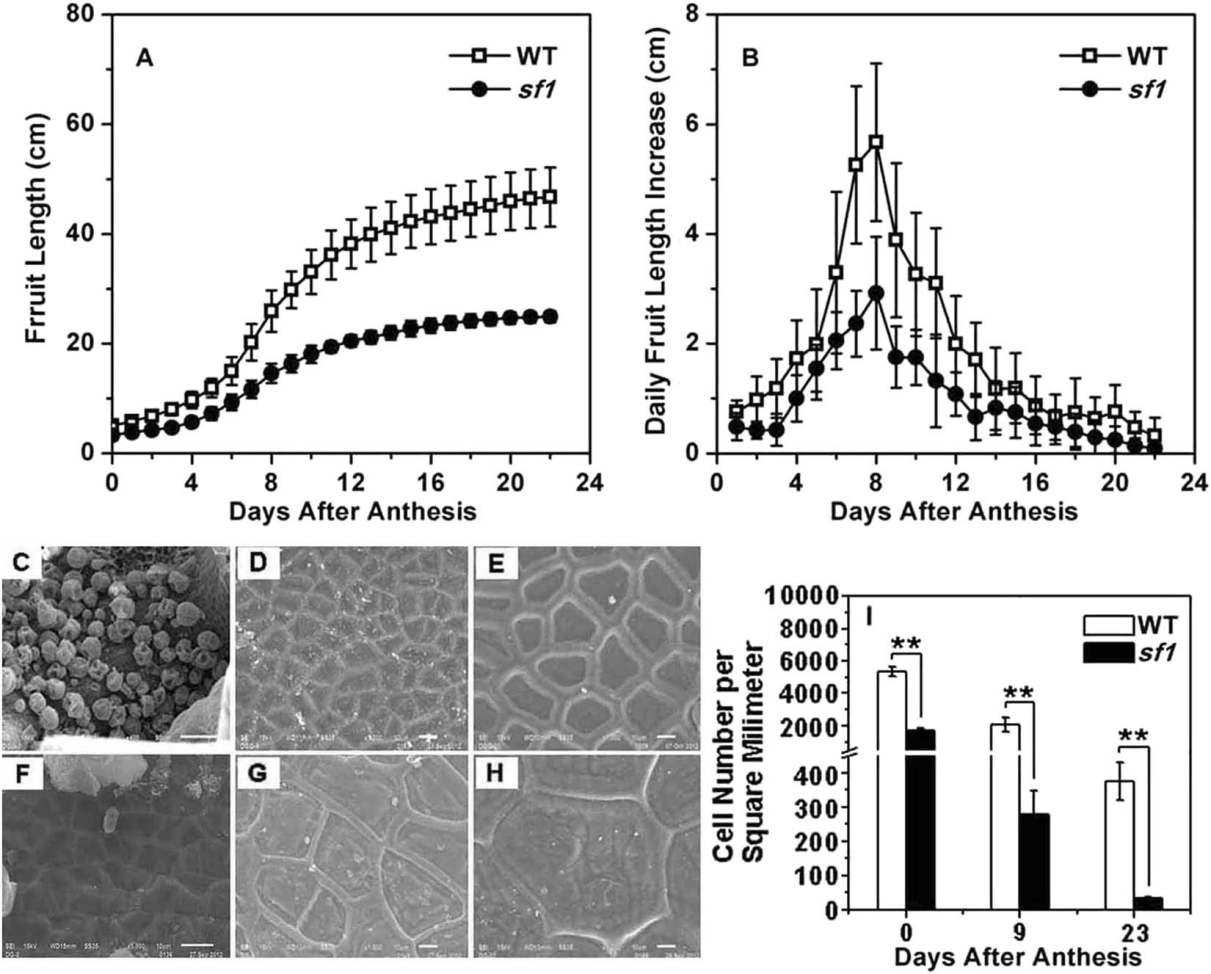
Kinematic analysis of fruit growth and pericarp cell size and number in WT and *sf1*. Fruit length (**A**) and daily increase in fruit length (**B**) were determined in WT and *sf1* from 0 to 22 DAA. Pericarp cell size of WT (**C**–**E**) and *sf1* (**F–H**) at 0 (C and F), 9 (**D** and **G**) and 23 DAA (E and H). Cell number per square millimetre (**I**) of WT and *sf1* pericarp was measured at three time points. ‘**’ indicates significant difference from WT at the 0.01 probability level. Scale bars represent 50 μm in (**C**) and 10 μm in (**D**–**H**). Vertical bars represent standard deviation (n = 18 for A and B, and n = 6–70 for **I**).

Kinematic analysis of fruit growth revealed that *sf1* fruits display a growth trend similar to that of WT (Fig. 2A) but undergo much slower growth throughout the entire growing phase (Fig. 2B). As a result, the difference in fruit length between *sf1* and WT continuously increased; at the end of the investigation, the fruit length of *sf1* was only approximately half of the WT fruit length (Fig. 1A).

To illuminate the cellular mechanism underlying the short fruit of *sf1*, we measured the cell number and cell size in the pericarp of WT and *sf1* fruits at 0, 9 and 23 DAA. We found significantly larger cells and fewer cells per square millimetre in *sf1* than in WT (Fig. 2C–I). Similar differences were observed in the sarcocarp of WT and *sf1* fruits (Supplementary Figure 3). These results indicate that the decrease in fruit length associated with *sf1* is due to a decrease in cell number.

### Fine mapping of *sf1*

**Figure 3:**
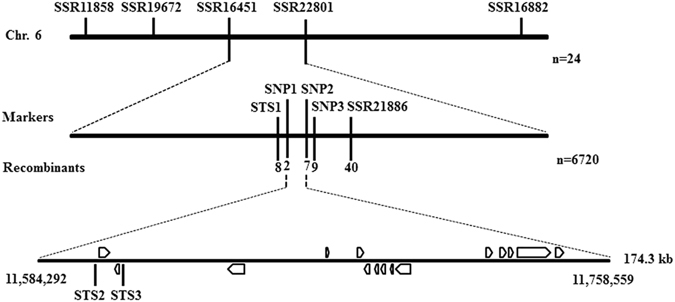
Mapping of the *sf1* gene. The *sf1* gene was fine-mapped to a 174.3 kb region between markers SNP1 and SNP2 on chromosome 6 in an F2 population containing 6,720 individuals from a cross between *sf1* and ‘Chinese long’ 9930.

To genetically map the *sf1* locus, an F2 population was first constructed by crossing *sf1* with ZG, a European greenhouse-type inbred line with short, light green, non-tuberculate fruits. Thirty-five polymorphic simple sequence repeat (SSR) markers were used in 24 F2 plants for rough mapping. The *sf1* locus was localized to a 4,456 kb region between SSR16451 and SSR22801 on chromosome 6 (Fig. 3). Because of the similarity in fruit size between *sf1* and ZG, it was difficult to distinguish mutant-phenotype plants from ZG plants in the F2 population. Therefore, we developed another F2 population from a cross between *sf1* and ‘Chinese long’ 9930 that has fruits similar to those of the WT parental line of *sf1*. All F1 progeny showed a long-fruit phenotype similar to that of WT. In the F2 population, long- and short-fruit plants segregated in a 3:1 ratio (79 long vs. 19 short; *x* ^*2*^ = 1.65 < *x* ^*2*^_*0*.*05*_ = 3.84; *p* > 0.05), demonstrating that the short-fruit phenotype of the *sf1* mutant is controlled by a single recessive nuclear gene. Two markers, STS1 (sequence-tagged site 1) and SSR21886, were then used to screen the F2 population derived from the cross between *sf1* and ‘Chinese long’ 9930, and 33 recombinant individuals were selected from 6,720 F2 plants (Supplementary Table 1). These recombinant individuals were transplanted into a solar greenhouse for fine mapping of *sf1* using three additional markers (SNP1, SNP2 and SNP3). The *sf1* locus was finally localized to an approximately 174.3 kb region (from 11,584,292 to 11,758,559) between markers SNP1 and SNP2 (Fig. 3). In this region, 15 annotated genes (*Csa6G176930*, *Csa6G176940*, *Csa6G177440*, *Csa6G178440*, *Csa6G178940*, *Csa6G178950*, *Csa6G179450*, *Csa6G179460*, *Csa6G179470*, *Csa6G179480*, *Csa6G180980*, *Csa6G180990*, *Csa6G181000*, *Csa6G181500* and *Csa6G181510*) were identified. The total genomic size of these 15 annotated genes was only 37.4 kb, and the remaining 136.9 kb was intergenic. The 15 annotated genes together with most of the non-coding region in the 174.3 kb region were analysed by sequencing, but no variation between *sf1* and WT (CNS2) was detected. Some partial segments (approximately 5.8 kb) in the 174.3 kb region were not sequenced because they could not be successfully amplified by PCR. We further detected the expression levels of these 15 annotated genes by semi-quantitative RT-PCR, and 12 of the 15 genes displayed no difference in expression level compared with that in WT (Supplementary Figure 4). The expression of the remaining three genes (*Csa6G180980*, *Csa6G180990* and *Csa6G181500*) was not detected, possibly due to low or no expression. This evidence indicates that there might be an unannotated gene or unknown regulatory element in this region. At the same time, we also identified two STS markers (STS2 and STS3) closely associated with the *sf1* locus (Fig. 3) that can be used in future cucumber breeding.

### Gene expression analysis in *sf1* fruits at different developmental stages

To investigate the molecular basis underlying the changes in fruit traits in *sf1*, comparative transcriptomic analysis of cucumber fruits was performed at three time points representing key developmental stages of cucumber fruit [cell proliferation (6 DBA), fruits sampled from *sf1* and WT; transition from slow to fast growth of cucumber fruit (2 DBA) and fastest growth (9 DAA), fruits sampled from the F2 population of a cross between *sf1* × ‘Chinese long’ 9930],which was based on our preliminary investigation and a previous study^18^. High-throughput RNA sequencing (RNA-Seq) produced 51, 167 and 128 million reads at these time points, respectively, for the mutant pools and 50, 117 and 66 million reads for the WT pools (Supplementary Table 2). After trimming low-quality reads and adapter sequences with Cutadapt (v.1.6) using a Cutoff Quality Score of 30, 47, 155 and 116 million reads from the mutant pools and 46, 109 and 55 million reads from the WT pools were successfully mapped to the cucumber genome (http://www.icugi.org/cgi-bin/ICuGI/genome/home.cgi?ver=2&organism=cucumber&cultivar=Chinese-long) and combined with known gene annotations (cucumber_ ChineseLong_v2.gff3) using the improved version of Tophat2 (Supplementary Table 2). All transcripts longer than 200 nt were used to predict the coding potential with the UniRef90 database; the genes with absolute values for logFC > 1 and *q*-value < 0.05 were defined as differentially expressed genes (DEGs) between the WT groups and the﻿ mutant groups using the R (v.3.1.0) language with the edgeR package. We found 266 DEGs, of which 173 were up-regulated and 93 were down-regulated in the mutant compared to WT at 6 DBA (Supplementary Figure 5). At 2 DBA, 263 DEGs were obtained; of these, 82 were significantly up-regulated and 181 were significantly down-regulated (Supplementary Figure 5). At 9 DAA, 478 DEGs were identified, 200 of which were up-regulated and 278 of which were down-regulated (Supplementary Figure 5). A Venn diagram (Supplementary Figure 5) shows that there was less DEG commonality between the 6 and 2 DBA and the 9 DAA age groups. To further confirm the DEGs identified by RNA-Seq analysis, quantitative PCR (qPCR) was performed with total RNA from *sf1* and WT fruits to test the variation in expression of 35 randomly selected genes (Supplementary Table 3). The genes whose variations in expression were tested by qPCR displayed the same expression patterns as in RNA-Seq analysis (Supplementary Table 3), indicating that the RNA-Seq results were reliable.

**Figure 4:**
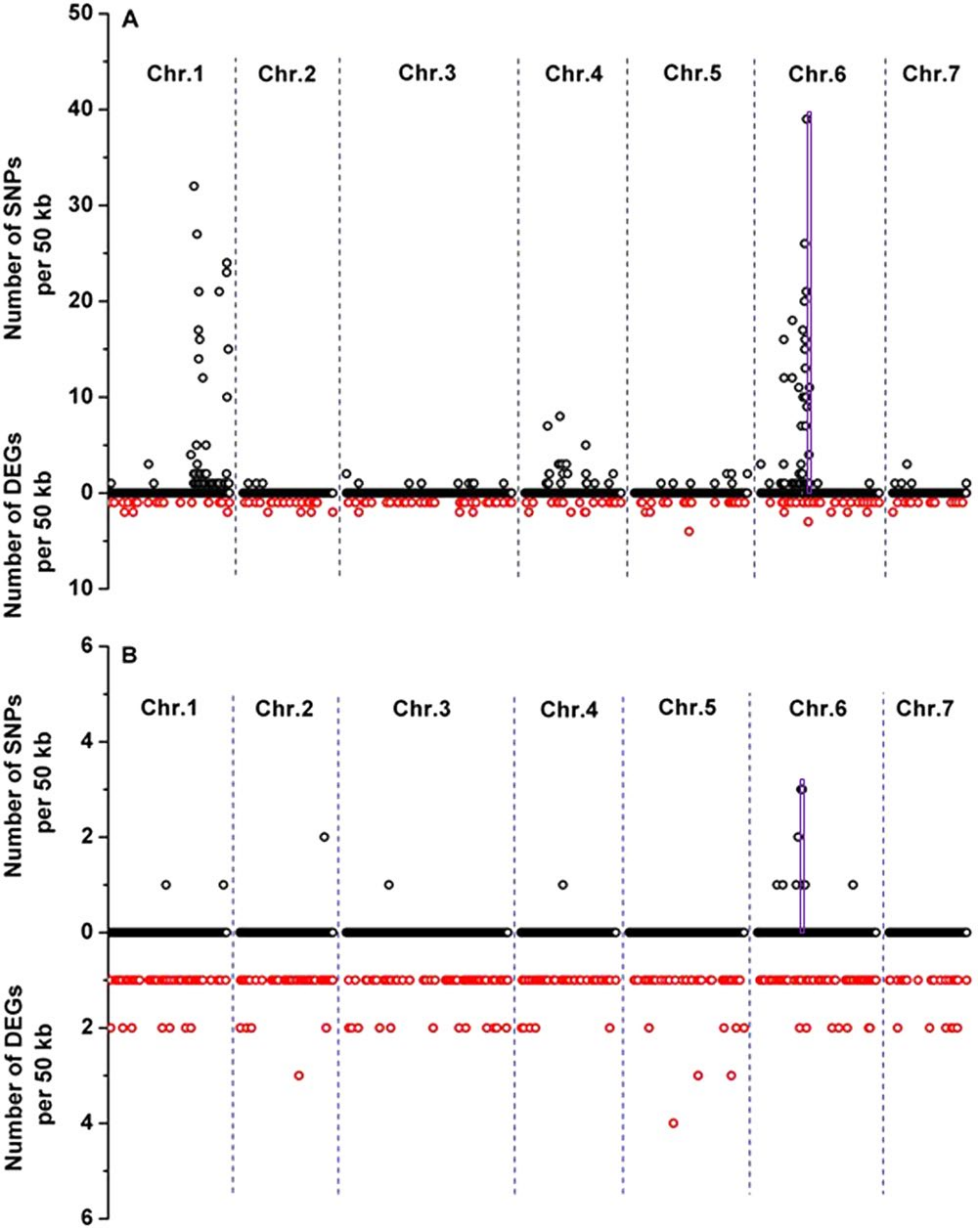
Chromosomal distributions of SNPs and DEGs in the cucumber genome. The SNPs and DEGs of all genes expressed in the fruit samples of the mutant vs. WT groups at 2 DBA (**A**) and 9 DAA (**B**), which came from the F2 population from a cross between *sf1* and ‘Chinese long’ 9930, are shown as the up direction (○) and the down direction (○) from the zero point on the same y-axis, respectively. The purple rectangles indicate the overlapping region (11,603,968 to 11,631,493) between the fine-mapped 174.3 kb region and the SNP-enriched region on chromosome 6. The number of SNPs and DEGs was calculated based on non-overlapping 50-kb bins.

Furthermore, SNPs were identified using the SAM tools, and variants with QUAL values higher than 20 were filtered as SNPs with high confidence. SNPs that were closely associated with genotype were further identified through Fisher’s exact test with *p*-value < 0.01. SNP-rich regions were identified on chromosomes 1 and 6 (from 10,053,234 to 11,631,603) at 2 DBA and on chromosome 6 (from 11,603,968 to 11,631,493) at 9 DAA in fruits of the mutant groups compared to the WT groups (Fig. 4). The SNP-enriched region on chromosome 6 was detected at both of the investigated time points and overlapped (from 11,603,968 to 11,631,493) with the region to which the *sf1* gene was fine-mapped (Fig. 4).

**Figure 5:**
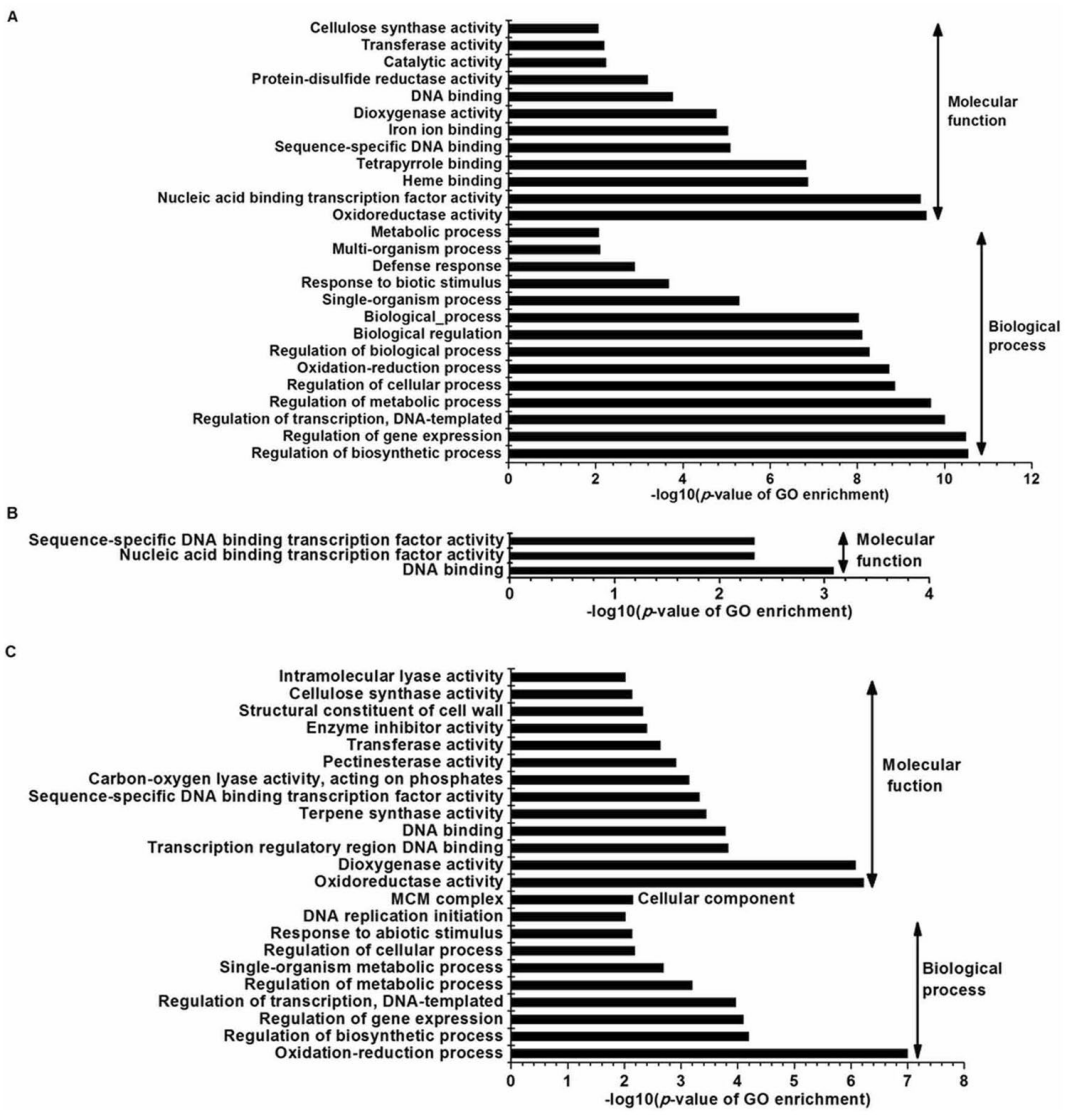
Significantly enriched GO terms (*p*-value < 0.01) in the cucumber fruits of the mutant vs. WT groups at 6 DBA (**A**), 2 DBA (**B**) and 9 DAA (**C**). GO terms were categorized into biological processes, cellular components and molecular functions based on their *p*-values.

**Figure 6:**
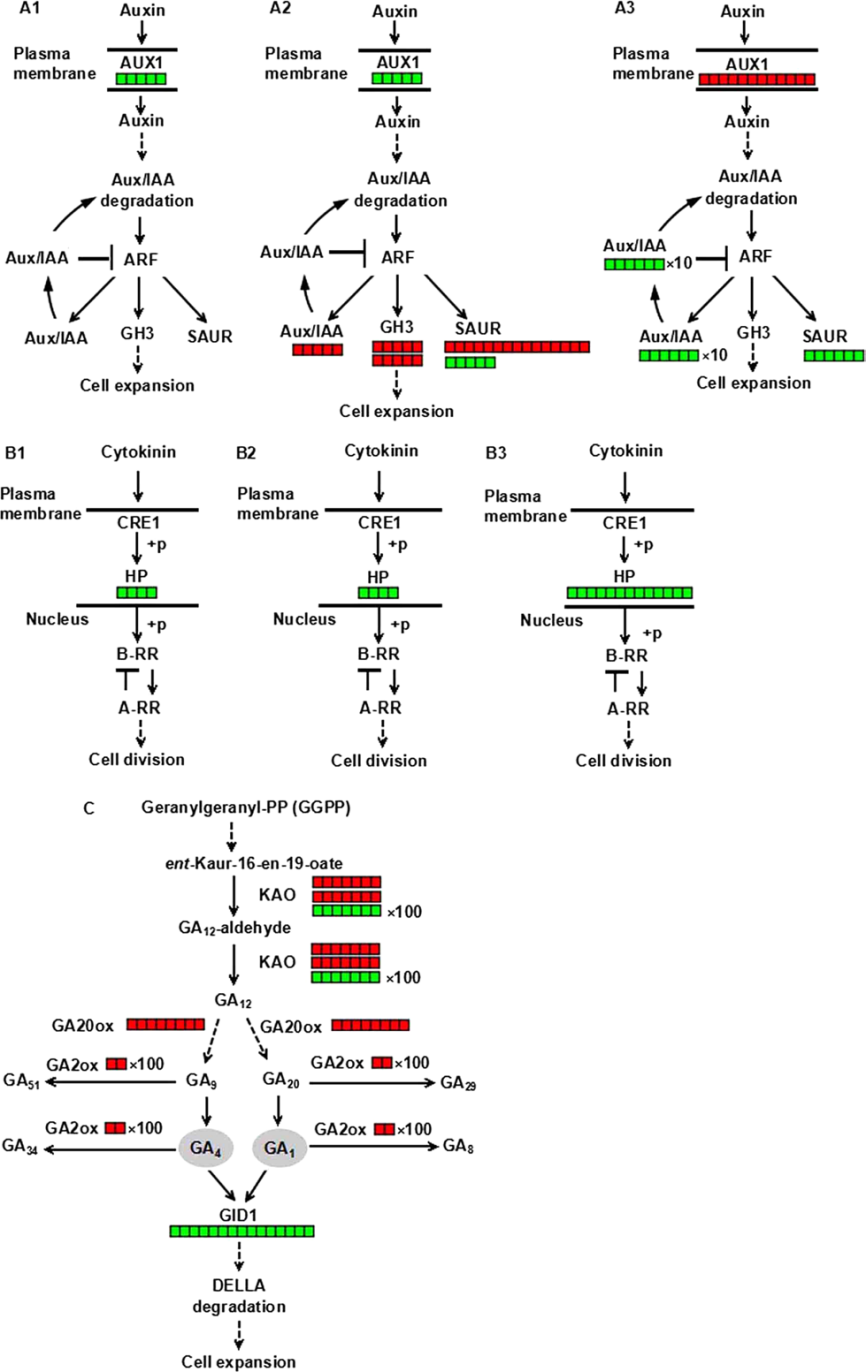
DEG-enriched pathways from the KEGG analysis. (**A**) DEG-enriched auxin signalling in the cucumber fruits of the mutant vs. WT groups at 6 DBA (A1), 2 DBA (A2) and 9 DAA (A3). (**B**) DEG-enriched cytokinin signalling in the cucumber fruits of the mutant vs. WT groups at 6 DBA (B1), 2 DBA (B2) and 9 DAA (B3). (**C**) DEG-enriched gibberellin biosynthesis and signalling in the cucumber fruits of the mutant vs. WT group at 9 DAA. The red closed rectangles represent the genes that were up-regulated, and the green closed rectangles represent the down-regulated genes. The number of rectangles indicates the fold gene expression difference between the mutant and WT fruits. Aux/IAA, Auxin/indole-3-acetic acid protein; ARF, auxin-response factor; SAUR, small auxin up RNA; CRE1, cytokinin response 1; HP, histidine phosphotransfer protein; B-RR, type-B response regulator; A-RR, type-A response regulator; KAO, ent-kaurenoic acid oxidase; GA, gibberellin; GA20ox, GA 20-oxidase; GA2ox, GA 2-oxidase; GID1, gibberellin insensitive dwarf 1.

To determine the functions of these DEGs, we performed gene ontology (GO) term enrichment analysis (*p*-value < 0.01) for the significantly influenced genes in fruits of the mutant groups at 6 DBA, 2 DBA and 9 DAA compared to the WT groups. The most significantly enriched GO terms were ‘Regulation of biosynthetic process’, ‘Regulation of gene expression’ and ‘Regulation of transcription, DNA-templated’ in the biological process group (Fig. 5). Only ‘DNA binding’ in the molecular function group was observed in mutant fruits at all three investigation time points (Fig. 5). KEGG analysis of these DEGs using a hyper-geometric distribution test revealed pathways that were closely associated with fruit phenotype and showed significant differences in fruits of mutant vs. WT groups. At 6 DBA, the significantly influenced genes included *AUX1*, which is involved in auxin signalling (Fig. 6A1), and *HP*, which is involved in cytokinin signalling (Fig. 6B1). At 2 DBA, the significantly influenced genes included *AUX1*, *Aux/IAA*, *GH3* and *SAUR*, which are involved in auxin signalling (Fig. 6A2), and *HP*, which is involved in cytokinin signalling (Fig. 6B2). At 9 DAA, the significantly influenced genes included *AUX1*, *Aux/IAA* and *SAUR* (Fig. 6A3), *HP* (Fig. 6B3), and *KAO*, *GA20*_*ox*_, *GA2*_*ox*_ and *GID1*, which is involved in gibberellin biosynthesis and signal transduction (Fig. 6C). At the same time, significant differences in the content of IAA, zeatin and GA_3_ were observed in *sf1* fruits compared with WT fruits (Supplementary Table 4). In addition, *BGLU*, *COMT*, *4CL*, *REF1*, *UGT72E* and *Peroxidase*, which are known to be in the phenylpropanoid biosynthesis pathway, and *CrtR*-*b*, *β*-*Carotene*-*isomerase*, *CrtZ*, *CCD7* and *NCED* in the carotenoid biosynthesis pathway, were significantly influenced at 9 DAA (Supplementary Figure 6; Supplementary Figure 7). These results provide molecular evidence for the phenotypic variations in *sf1* fruits.

**Figure 7:**
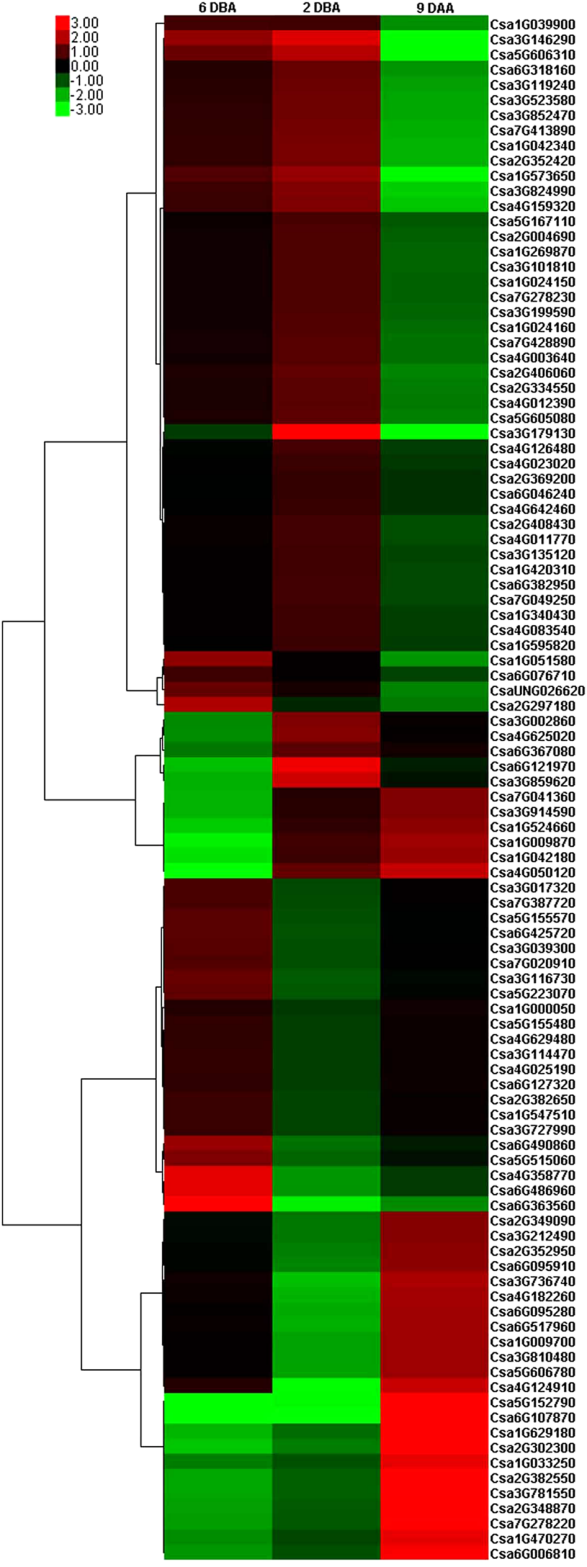
Heatmap showing the differential expression of selected transcriptional factors in the mutant vs. WT group fruits at 6 DBA, 2 DBA and 9 DAA. Genes highly or weakly expressed in the mutant vs. WT group fruits are shown in red and green, respectively. Genes without expression in the mutant vs. WT group fruits are shown in black. The heatmap was generated using cluster 3.0 software.

We also identified significantly differentially expressed transcription factors in fruits of the mutant vs. the WT groups. At 6 DBA, the expression of 29 transcription factors belonging to the following gene families was up-regulated: *AP2*/*ERF*, *GRAS*, *HSF*, *LFY*, *MADS*, *NAC*, *WRKY*, *YABBY* and *Zinc finger protein*; by contrast, the expression of 7 transcription factors belonging to the following gene families was down-regulated: *bZIP*, *MYB*, *NAC*, *TCP*, *WD40*, *WRKY* and *Zinc finger protein* (Fig. 7; Table 1). At 2 DBA, the expression of 12 transcription factors belonging to the following gene families was up-regulated: *bHLH*, *bZIP*, *MADS*, *MYB*, *WRKY*, *YABBY* and *Zinc finger protein*; by contrast, the expression of 13 transcription factors belonging to the gene families *AP2*/*ERF*, *bZIP*, *MADS*, *MYB*, *SBP*, *WRKY*, *YABBY* and *Zinc finger protein* was down-regulated (Fig. 7; Table 1). At 9 DAA, there were 10 up-regulated transcription factors belonging to the gene families *AP2*/*ERF*, *HSF*, *MYB*, *NAC*, *YABBY*, and *Zinc finger protein* and 39 down-regulated transcription factors belonging to the gene families *AP2*/*ERF*, *bHLH*, *bZIP*, *HSF*, *MYB*, *NAC*, *NF*-*YA*, *WRKY*, and *Zinc finger protein* (Fig. 7; Table 1). These results suggest that transcription factors might play a critical role in determining fruit length.

**Table 1:** List of transcriptional factors that were expressed differentially in cucumber fruits of mutant vs. WT groups.

Sampling time	IPR annotation	Gene number	Gene ID
6 DBA	AP2/ERF	5	*Csa3G017320 Csa3G114470 Csa3G116730 Csa5G155570 Csa6G490860*
6 DBA	bZIP	1	*Csa7G041360*
6 DBA	GRAS	1	*Csa3G039300*
6 DBA	HSF	2	*Csa5G155480 Csa7G387720*
6 DBA	LFY	1	*Csa1G000050*
6 DBA	MADS	6	*Csa1G039900 Csa1G051580 Csa3G179130 Csa4G126480 Csa4G358770 Csa6G076710*
6 DBA	MYB	1	*Csa1G524660*
6 DBA	NAC	5	*Csa1G009870 Csa4G629480 Csa6G127320 Csa6G425720 Csa7G020910*
6 DBA	TCP	1	*Csa1G042180*
6 DBA	WD40	1	*Csa3G914590*
6 DBA	WRKY	8	*Csa1G547510 Csa2G297180 Csa2G382650 Csa3G727990 Csa4G050120 Csa5G223070 Csa6G363560 Csa6G486960*
6 DBA	YABBY	1	*CsaUNG026620*
6 DBA	Zinc finger protein	3	*Csa1G033250 Csa4G025190 Csa5G515060*
2 DBA	AP2/ERF	1	*Csa2G349090*
2 DBA	bHLH	1	*Csa3G002860*
2 DBA	bZIP	2	*Csa3G810480 Csa4G625020*
2 DBA	MADS	8	*Csa1G039900 Csa1G051580 Csa3G179130 Csa4G126480 Csa4G182260 Csa6G076710 Csa6G095280 Csa6G367080*
2 DBA	MYB	5	*Csa1G009700 Csa2G352950 Csa3G736740 Csa6G095910 Csa6G121970*
2 DBA	SBP	1	*Csa6G517960*
2 DBA	WRKY	2	*Csa2G297180 Csa3G212490*
2 DBA	YABBY	2	*Csa5G606780 CsaUNG026620*
2 DBA	Zinc finger protein	3	*Csa1G033250 Csa3G859620 Csa4G124910*
9 DAA	AP2/ERF	7	*Csa1G269870 Csa1G340430 Csa2G382550 Csa3G135120 Csa4G023020 Csa5G167110 Csa6G318160*
9 DAA	bHLH	4	*Csa1G024150 Csa1G042340 Csa2G369200 Csa7G428890*
9 DAA	bZIP	2	*Csa1G573650 Csa3G119240*
9 DAA	HSF	3	*Csa1G629180 Csa2G004690 Csa3G146290*
9 DAA	MYB	7	*Csa1G024160 Csa2G302300 Csa2G408430 Csa3G199590 Csa5G152790 Csa6G046240 Csa7G413890*
9 DAA	NAC	9	*Csa2G334550 Csa3G101810 Csa3G523580 Csa3G824990 Csa3G852470 Csa4G011770 Csa5G606310 Csa6G107870 Csa6G382950*
9 DAA	NF-YA	1	*Csa4G159320*
9 DAA	WRKY	2	*Csa2G406060 Csa4G012390*
9 DAA	YABBY	1	*Csa2G348870*
9 DAA	Zinc finger protein	13	*Csa1G420310 Csa1G470270 Csa1G595820 Csa2G352420 Csa3G781550 Csa4G003640 Csa4G083540 Csa4G642460 Csa5G605080 Csa6G006810 Csa7G049250 Csa7G278220 Csa7G278230*

## Discussion

Fruit size, one of the most important appearance quality parameters for vegetables such as cucumber and tomato, commonly displays high variation among cultivars. Many QTLs that control cucumber fruit length have been detected in different populations, developmental stages and growing seasons, and 14 were mapped on chromosome 6^5–7, 19–24^. Twelve of the 14 QTLs on chromosome 6 were named *FL6*.*1* (or *MFL6*.*1*or *Sfl6*.*1*), but their positions differed. In addition to five QTLs, *qFL6*.*1* and *qMFL6*.*1* detected by Weng *et al*., *Sfl6*.*1* and *Sfl6*.*2* detected by Miao *et al*., and *fl6*.*1* detected by Yuan *et al*. (only their genetic positions are available: from 43.5 to 77.3 cM, from 43.5 to 54.3 cM, from 45.7 to 49.7 cM, from 49.7 to 52.4 cM, and from 183.2 to 186.9 cM, respectively)^6, 19, 20^, the remaining nine QTLs’ physical positions have been detailed as follows: from 9.245 to 10.817 Mb and from 10.816 to 11.520 Mb for two *fl6*.*1s* detected by Bo *et al*. under two different growing conditions, respectively^5^; from 12.488 to 12.711 Mb for *fl6*.*1* detected by Wei *et al*.^21^; from 12.931 to 19.653 Mb for *fl6*.*1* detected by Wang *et al*.^22^; from 15.630 to 22.271 Mb for *MFL6*.*1*detected by Weng *et al*.^6^; from 23.205 to 23.755 Mb for *fl6*.*1* detected by Qi *et al*.^23^; from 23.544 to 24.578 Mb for *fl6*.*1* detected by Cheng *et al*.^24^; and from 24.289 to 27.896 Mb for *fl6*.*1* and *fl6*.*2* detected by Miao *et al*.^19^. In addition, Jiang *et al*. identified a fruit length-related locus in the region from 18.1 Mb to 19.0 Mb on chromosome 6 through SNP association analysis based on transcriptomic data^7^. However, none of the regions in which these reported QTLs/genes were localized overlaps with the region to which the *sf1* gene was mapped (from 11.584 to 11.759 Mb) on chromosome 6, indicating that *sf1* might be an uncharacterized locus.

We further analysed the 174.3 kb region in which *sf1* was mapped using PCR-based sequencing and found no difference in the 168.5 kb sequenced region between *sf1* and WT. In addition, there were no differences in expression among the genes in this region (Supplementary Figure 4). Therefore, the responsible mutation is still unknown. This discrepancy might be explained with the following two possibilities. First, in the candidate region, 5.8 kb segments (if no gap) have not yet been sequenced due to technical difficulties, thus, we do not know whether there are mutations or gaps in the un-sequenced region. In fact, there are still many un-assembled scaffolds in the cucumber genome. In this study, we also found that there are 9 DGEs at 6 DBA, 64 DGEs at 2 DBA and 10 DGEs at 9 DAA mapped on un-assembled scaffolds. Second, epigenetic differences might be responsible for the mutation. Variations such as DNA methylation might occur at the epigenetic level, which could influence gene expression either inside or outside the mapped regions because of the complexity of cis-acting regulatory DNA elements^25–27^. Further studies should address this problem in the future.

It has been well demonstrated that cell division and cell enlargement play critical roles in final fruit length, diameter and yield. To illuminate how *sf1* regulates fruit length, we analysed *sf1* fruits using histological, physiological and comparative transcriptomic approaches. We found that the cell number of *sf1* fruits was significantly lower than that of WT fruits (Fig. 2I). Comparative transcriptomic analysis revealed that a cucumber orthologue of *Arabidopsis* histidine phosphotransfer proteins (AHPs), which are a critical component of plant cytokinin signalling, was significantly repressed at three investigated time points in the mutant group fruits compared to its expression in the WT group fruits (Fig. 6B). AHPs belong to a protein family that contains five members, AHP1-AHP5; these proteins are required for transfer of a phosphoryl group from the *Arabidopsis* histidine kinase (AHKs) to *Arabidopsis* response regulators (ARRs)^28^. To date, all reported results on AHPs suggest that, with the exception of AHP4^28^, they function positively in cytokinin signalling. Mutations in these positive AHPs can cause a series of developmental abnormalities, such as shortened primary roots, reduced vascular development, decreased fertility, increased numbers of embryos and seeds, and adventitious root development^29^. It has been demonstrated that cucumber fruit cell division primarily occurs prior to anthesis and that the rate of cell division then gradually decreases until 5 days post pollination (DPP)^30, 31^. The significantly decreased expression of *histidine phosphotransfer protein* (*HP*) in *sf1* fruits during cucumber fruit development provides a possible molecular explanation for the dramatic reduction in cell number, the main reason for the short-fruit phenotype of *sf1*.

Although some genes responsible for gibberellin biosynthesis and signalling were stimulated, the expression of GID1 (gibberellin insensitive dwarf 1) was significantly lower in *sf1* fruits at 9 DAA (Fig. 6C). GID1 is a gibberellin receptor that can stimulate the degradation of DELLA in plants through a ubiquitin-proteasome pathway^32^. DELLA proteins are repressors of GA signalling and can restrict GA-mediated plant growth and development, including fruit development^33^. Binding of GA to GID1 can facilitate GID1-DELLA interaction and thus stimulate the degradation of DELLAs^34^. It has been reported that cucumber fruit elongation occurs most rapidly between 4 DPP and 12 DPP and that the peak rate of elongation occurs at approximately 8 DPP^18, 35^. The significant reduction in GID1 expression at 9 DAA (Fig. 6C) might block fruit elongation at a key stage and thus exacerbate the shortening of fruit length in *sf1*.

Previous studies have proposed a positive relationship between cell size and fruit length in cucumber^35^. However, in this study, we observed that although *sf1* fruits displayed dramatically decreased fruit length and decreased daily growth (Fig. 2A and B), cell size in *sf1* fruits was significantly increased relative to that in WT fruits, possibly due to abnormalities in auxin signalling in *sf1* fruits. In particular, at 2 DBA the expression of key auxin-responsive genes was significantly higher in the mutant group fruits than in the WT group fruits from the F2 population (Figs 2C–H and 6A). The significantly larger cell size in *sf1* fruits might be a compensatory response to the decrease in cell number. Similar effects have been reported in cucumber﻿,﻿ in which a decrease in cell number is compensated for by an increase in cell size when assimilated supply is limited^36^; in *Arabidopsis* mutants that affect leaf size by producing decreased cell numbers but increased cell size^37^; in *Arabidopsis* mutants that affect integument development by causing increased cell division and reduced cell size^38^; and in the *Antirrhinum majus* floral mutant *formosa* (*fo*), which has increased petal size but smaller cells^39^. The abnormal cell size might also be an important reason for the increased cracking of *sf1* fruits (Fig. 1B and 2C–H). At the same time, we observed significant effects of the *sf1* mutation on the expression of transcription factors that play critical roles in plant hormone signalling (Fig. 7; Table 1). These up- or down-regulated transcription factors might bridge the three signalling pathways of cytokinin, gibberellins and auxin to co-regulate fruit length in the *sf1* mutant. Tan *et al*. also reported that the transient balance of endogenous hormones might play a key role in the regulation of cucumber fruit length^40^.

In addition to the significant decrease in fruit length (Fig. 1A), significant changes in other fruit-related traits also occurred in the *sf1* mutant (Fig. 1B–J), indicating that *sf1* has pleiotropic effects. Genes with pleiotropic effects have been widely identified and well characterized in previous studies. For example, a rice mutant losing *FISH BONE* (*FIB*) gene function, an orthologue of *TRYPTOPHAN AMINOTRANSFERASE OF ARABIDOPSIS* (*TAA*) genes, has pleiotropic abnormal phenotypes, including decreased leaf size with larger lamina joint angles, smaller panicles, abnormal vascular development and organ identity, defects in root development and reduced endogenous IAA levels^41^. In *Populus trichocarpa*, an orthologue of the class III homeodomain-leucine zipper transcription factor gene *REVOLUTA* has been demonstrated to be significantly associated with poplar fungal and rust resistance, leaf abscission, cellulose content, and auxin signalling^42^. In cucumber, a putative *R2R3*-*MYB* transcription factor gene has been shown to control both black spine and orange mature fruit colours of cultivated cucumber, thus displaying pleiotropic effects^43^.

Comparative transcriptomic analysis provided some molecular evidence for the pleiotropic effects of the *sf1* gene as well. For example, fruit hardness and crispness are mainly dependent on the composition of the fruit’s cell walls^15–17^; thus, changes in the content of cell wall components might result in variations in fruit hardness and crispness. In *sf1* fruits, significantly higher lignin content was observed (Fig. 1M), consistent with the observed significant stimulation of the lignin biosynthesis pathway (Supplementary Figure 6; Supplementary Table 5). This might be the reason *sf1* fruits display greater fruit hardness and crispness. Additionally, *sf1* fruits were shown to have significant differences in carotenoid biosynthesis (Supplementary Figure 7; Supplementary Table 5), providing molecular evidence for *sf1*-mediated regulation of carotenoid content in cucumber fruits (Supplementary Figure 2I). However, full explanation of other altered traits such as spine morphology, stigma and seed cavity size in *sf1* requires further study.

In summary, the results of the present study demonstrated that *sf1* is a pleiotropic effector of the external and internal qualities of cucumber fruits. Altered expression of genes in the auxin, cytokinin signalling and GA biosynthesis and signalling pathways might be the reason for the decrease in cell number in the *sf1* mutant, which led to a decrease in fruit length. Future studies, such as cloning and functional analysis of *sf1*, are needed to better understand the molecular mechanisms involved in the regulation of cucumber fruit growth and development.

## Materials and Methods

### Plant materials and growth conditions

Cucumber (*Cucumis sativus* L., 2n = 14) inbred line CNS2 (WT, North China type), ZG (North European type), ‘Chinese long’ 9930 (North China type), and the *sf1* mutant were used. Plants were grown for two generations each year from 2011 to 2016 in the greenhouse of the experimental field at Shandong Agricultural University. Standard management was performed during the cultivation period.

### Phenotype investigation

Fruit length (FL) was measured from 0 DAA to 22 DAA. Daily fruit growth (DFG) was calculated based on the following formula: DFG = FL_n_ − FL_(n−1)_, where FL_n_ and FL_(n−1)_ represent fruit length at n and n−1 DAA (1 ≤ n ≤ 22). Each parameter was determined from 18 biological repeats.

### Fruit quality analysis

Cucumber fruits from WT and *sf1* were sampled at 0, 9 and 23 DAA, and their hardness and crispness were measured using a texture analyser (TA.XT Plus, Stable Micro Systems Ltd) with a P/2N and the following settings: 3 mm s^−1^ pre-test speed, 1 mm s^−1^ test speed, 0.5 mm s^−1^ post-test speed, 5 mm penetration depth and 10 g minimum trigger force.

WT and *sf1* fruits were sampled at 9 DAA and dried at 55 °C for 48 h. The dried samples were weighed and ground to pass a 1-mm sieve. Subsequent analysis of acid-soluble lignin content in the samples was performed according to the previously described method^44^ using a modification for amylase^45^.

Three biological repeats were used for the determination of each parameter.

### Microscopic investigation of *sf1* fruits

Pericarp and sarcocarp cell size in WT and *sf1* fruits were investigated using a scanning electron microscope (SEM) at 0, 9 and 23 DAA according to the method previously described^46^. After 24-h fixation in 2.5% (w/v) glutaraldehyde at 4 °C, the pericarp and sarcocarp samples were washed three times with full-strength phosphate- buffered saline (PBS) and further processed with 1% (v/v) OsO4. The OsO4-processed samples were dehydrated through six volume concentrations of ethanol (30%, 50%, 70%, 80%, 90% and 100%) three times, critical- point-dried with liquid CO_2_ and coated with gold palladium (EIKO IB-3). The resulting samples were viewed under a SEM (JSM-6610LV). Cell number per unit area of pericarp was then calculated using Image J software (v 2.1.4.7, NIH).

Stigmas from WT and *sf1* at 0 DAA and spines of WT and *sf1* fruits at 11 DAA were investigated under a dissection microscope (LEICA M165 FC).

### Fine mapping of the *sf1* gene

For mapping the *sf1* locus, the *sf1* mutant was crossed with ZG and ‘Chinese long’ 9930 to construct F2 segregation populations. Thirty-five SSR markers were used to perform preliminary mapping of *sf1* with the F2 population from the cross between *sf1* and ZG. In total, 6,720 F2 plants from the cross between *sf1* and ‘Chinese long’ 9930 were further analysed with 22 SNP markers for fine mapping of *sf1*. The PCR products were checked by ethidium bromide (EB) staining under UV light following separation on agarose gels. All primers used for mapping are listed in Supplementary Table 6.

### Construction of RNA libraries for comparative transcriptomic analysis

Transcriptomic profiling was analysed using cucumber fruits from WT and *sf1* at 6 DBA and from the F2 population from *sf1* × ‘Chinese long’ 9930 at 2 DBA and 9 DAA. The middle part of the fruits at all three stages was used to extract total RNA. The F2 population from *sf1* × ‘Chinese long’ 9930 was used at the two later time points to test the fine mapping results obtained through SNP association analysis. Thirty individual plants were used for fruit sampling; these fruits were then pooled as one biological sample for WT and mutant phenotyping. mRNA was isolated from total RNA using oligo-dT magnetic beads and fragmented with fragmentation buffer for cDNA synthesis. After the addition of adenine, the resulting cDNAs were linked to adapters and purified by gel electrophoresis; finally, cDNAs approximately 250 bp in length were collected for PCR amplification. After quality checking and quantification with an Agilent 2100 Bioanalyzer and the ABI StepOnePlus Real-Time PCR System, all libraries were sequenced on the Illumina HiSeqTM 2000 platform at Honortech (Beijing, China).

### Bioinformatics analysis

Removal of low-quality and adaptor sequences from the raw data was performed with Cutadapt (v1.6)^47^. Clean sequencing data for each sample were aligned to the reference genome (http://www.icugi.org/cgi-bin/ICuGI/genome/home.cgi?ver=2&organism=cucumber&cultivar=Chinese-long) and combined with known gene annotations (cucumber_ChineseLong_v2.gff3) using the improved version of Tophat2 software (v 2.1.0)^48^ with the following parameters: read-mismatches 2, mate-inner-dist 50, mate-std-dev 80 and library-type fr-unstranded. Four procedures in Cufflinks^49^, including cufflinks, cuffmerge, cuffquant and cuffnorm, were used to reconstruct transcripts, identify novel transcripts, quantify transcripts and normalize expression values as FPKM (fragments per kilobase of transcript per million mapped reads).

CPC software with the UniRef90 database was used to predict the coding potential of all transcripts longer than 200 nt^50^. Transcripts scored greater than 0 were identified as encoding genes, and those scored less than 0 were considered non-coding genes. Functional annotation for expressed transcripts was performed using the BLASTX program (v 2.2.29) based on the NR, eggNOG and Swiss-Port databases with an e-value cutoff of <1e-5. Identification of domains and gene families and GO analysis were performed using InterProScan (v. 5.8–49.0). GO plant slim level 2 was used to obtain GO functional classifications for all transcripts. Mapping of the transcripts to KEGG pathways was performed using KOBAS 2.0^51^ with default parameters.

The R (v.3.1.0) language with the edgeR package was used to identify DEGs. The fold change (FC) between the two groups was calculated based on the following formula: logFC = log_2_ (mutant group/WT group). Genes that differed in expression between the mutant and WT groups were defined as DEGs in this study if the absolute value of the logFC was greater than 1 and the *q*-value was less than 0.05. A hypergeometric distribution test was carried out to identify GO functions and KEGG pathways in which the DEGs were significantly enriched with *p*-values < 0.01 in comparison to the total expressed transcripts. Python 2.7.5 with numpy (1.9.2), scipy (0.15.1) and matplotlib (1.4.3) was used to perform cluster analysis of the DEGs based on Ward’s method with Euclidean distance as a measurement of similarity.

Based on alignments by Tophat2, SNPs were identified using SAM tools (v. 0.1.19) with the set parameters of -Q13 and -Q20. Variants with QUAL values higher than 20 were filtered as SNPs with high confidence. Fisher’s exact test was used to further identity the SNPs associated with the genotype through the cutoff of *p*-value < 0.01, which was based on the principle of MutMap^52^.

## Electronic supplementary material

Supplementary information
