# Supplementary material for: Transcriptomic analysis of short-fruit 1 (sf1) reveals new insights into the variation of fruit-related traits in Cucumis sativus
Source: Sci Rep. 2017 Jun 7;7:2950. doi: 10.1038/s41598-017-02932-5 (PMC5462832; doi:10.1038/s41598-017-02932-5)
Supplement: Supplementary file 1 — Supplementary information [file 41598_2017_2932_MOESM1_ESM.pdf]

Transcriptomic analysis of *short-fruit 1* (*sf1*) reveals new insights into the variation of fruit-related traits in *Cucumis sativus*

Lina Wang[1], Chenxing Cao[1], Shuangshuang Zheng[1], Haiyang Zhang, Panjing Liu, Qian Ge, Jinrui Li, Zhonghai Ren[*]

State Key Laboratory of Crop Biology; Key Laboratory of Biology and Genetic Improvement of Horticultural Crops (Huanghuai Region), Ministry of Agriculture; College of Horticulture Science and Engineering, Shandong Agricultural University, No.61 Daizong road, Tai'an, Shandong 271018, China

1 Lina Wang, Chenxing Cao and Shuangshuang Zheng contributed equally to this work.

* To whom correspondence should be addressed. Email: zhren@sdau.edu.cn

Supplementary Materials and Methods

Phenotype investigation

To analyse the difference in phenotypes between WT and *sf1*, the plant height, which was defined as the length from the plant base to the top bud, and the stem diameter at the fourth node counted from the plant base were determined at flowering stage. The leaf area of the first and the second functional leaves, which were counted from the plant apex, was measured using the method described previously [1]. Eighteen biological repeats were used for statistical analysis.

Seed cavity and fruit diameter of WT and *sf1* were measured with a Vernier calliper at 23 DAA; 12 biological repeats were used for statistical analysis.

Fruit quality analysis

The chlorophyll and carotenoid contents of the pericarp and sarcocarp of WT and *sf1* fruits were determined at 11 DAA. Samples weighing 0.2 g each were used for chlorophyll and carotenoid extraction with 80% acetone (v/v). The absorbance of these extracts at 663, 647 and 470 nm was measuring using a spectrometer (UV-2450, Shimadzu, Kyoto, Japan). The concentration of chlorophyll and carotenoids in each extract was determined according to the methods of Ikan [2] and Strain and Svec [3]. The content of each pigment in fruit samples was calculated based on the following formula: Cp (mg g^{-1}) = (C′p * volume of extract * dilution factor) / fresh weight of sample, where Cp represents the content of chlorophyll or carotenoids in the sample and C′p represents the concentration of chlorophyll and carotenoids in each extract. Three biological repeats were used for the determination of each parameter.

Quantitative PCR

Total RNA was isolated from *sf1* and WT fruits at 6 DBA, 2 DBA and 9 DAA using the RNeasy Plant Mini Kit (QIAGEN) according to the manufacturer's instructions. First-strand cDNAs were prepared from the isolated total RNAs using the PrimeScript RT-PCR Kit (Takara). Primers were designed with the aid of Primer 5 software and synthesized by GENEWIZ (Beijing, China; Supplementary Table 7). qPCR was performed with three biological repeats and the cucumber *actin* gene as internal control on a ROCHE LightCycler® 480 real-time PCR System (Roche, Switzerland). The relative expression of genes was calculated according to the $2^{-\Delta\Delta Ct}$ method and is presented as the mean ± standard error.

Determination of hormone content in fruit

A liquid nitrogen-treated fruit sample weighing 0.3 g was freeze-dried in a freeze dryer and squashed well with a mortar and pestle. The sample was then transferred to a tube with 5 ml of extraction buffer (30 mg L^{-1} sodium diethyldithiocarbamate in pre-cooled 100% acetonitrile) and stored at 4 ℃ for at least 12 h. After centrifugation for 10 min at 8,000 rpm and 4 ℃, the supernatant was transferred to a glass bottle, and the pellet was re-extracted with 4 ml of extraction buffer on a shaker with agitation at 230 rpm for 2 h. The supernatant was collected in the glass bottle after centrifugation for 10 min at 8,000 rpm and 4 ℃. The pellet was washed with 2 ml of extraction buffer, and the supernatant was transferred to the glass bottle containing the supernatant from the previous centrifugation step. The obtained extract was dried in a distillation bottle at 38 ℃ under decreased pressure conditions, dissolved in a mixture of 2 ml of 0.4 M phosphate buffer saline (PBS) (pH 8.0) and 2 ml of chloroform, and transferred to a new glass bottle. The distillation bottle was washed with 3 ml of 0.4 M PBS followed by 2 ml of chloroform, both of which were also collected in the glass bottle. After further purification by chloroform and polyvinylpyrrolidone (PVPP), the resultant solution was used for hormone extraction with ethyl acetate. The hormone solution was dried at 36 ℃ and finally dissolved in 1 ml of mobile phase [a mixture of methanol and 0.04% (v/v) acetic acid at a volume ratio of 45 to 55] for the determination of hormone content by

LC-MS on a TSQ Quantum system (Thermo, Waltham, MA, USA) equipped with a Hypersil Gold C18 column at a flow rate of 3 µl min^{-1}, column temperature of 25 ℃, and 10 µl of input. Standard curves were prepared with IAA, zeatin and GA_3, respectively.

Supplementary references

1. Robbins, N.S. & Pharr, D.M. Leaf area prediction model for cucumber from linear measurements. *Hortic Sci* **22**, 1264-1266 (1987).

2. Ikan, R. Natural Products, A Laboratory Guide (Academic Press, 1969).

3. Strain, H.H. & Svec, W.A. Extraction, separation, estimation and isolation of the chlorophylls. In: The Chlorophyll (eds Vernon, L.P. *et al.*) 21-66 (Academic Press, 1966).

Supplementary Table 1: Phenotype analysis of recombinant individuals from F_2 population of *sf1* × 'Chinese long' 9930.

Recombinant individual	Genotype					Phenotype
	STS1 11,539,847	SNP1 11,584,292	SNP2 11,758,559	SNP3 11,763,286	SSR21886 12,093,103	
1	B	B	H	H	H	M
2	H	H	B	B	B	WT
3	B	B	B	B	H	M
4	B	B	B	B	H	M
5	B	B	B	B	H	M
6	B	B	B	B	H	M
7	B	B	B	B	H	M
8	B	B	B	B	H	M
9	B	H	H	H	H	WT
10	H	H	B	B	B	WT
11	B	B	B	B	H	M
12	B	H	H	H	H	WT
13	B	B	B	B	H	M
14	B	B	B	B	H	M
15	H	H	H	H	B	WT
16	B	B	H	H	H	WT
17	B	B	B	B	H	M
18	B	B	B	B	H	M
19	H	H	H	H	B	WT
20	B	B	B	B	H	M
21	B	B	B	B	H	M
22	H	H	H	H	B	WT
23	H	H	H	B	B	WT
24	H	H	H	H	B	WT
25	H	H	H	H	B	WT
26	B	H	H	H	H	WT
27	H	H	H	H	B	WT
28	B	B	B	B	H	M
29	H	H	H	H	B	WT
30	B	B	H	H	H	M
31	B	B	H	H	H	M
32	B	B	B	B	H	M
33	H	H	H	H	B	WT

Note: B represents *sf1* genotype; H represents heterozygous genotype; M represents *sf1* phenotype; WT represents wild type phenotype.

Supplementary Table 2: Throughput of RNA-Seq.

Sampling time	Samples	Total reads number	Total mapped reads	Mapped	Q20 percentage	GC percentage
6 DBA	wild	49,457,340	45,585,911	92.17%	95.29%	43.00%
	mutation	50,897,300	46,793,050	91.94%	95.86%	42.50%
2 DBA	wild	117,083,563	108,577,621	92.74%	99.76%	43.99%
	mutation	167,198,033	154,568,477	92.45%	99.76%	43.60%
9 DAA	wild	66,284,929	55,329,684	83.47%	99.22%	48.94%
	mutation	128,175,463	116,201,536	90.66%	99.71%	48.53%

Supplementary Table 3: Verification of gene expression in *sf1* vs. WT fruits by qPCR.

Gene ID	Sampling time	log_2(mutant/ WT) from RNA-seq	log_2(mutant/WT) from qPCR	Gene expression up- or down-regulated
Csa2G264590	6 DBA	-2.34	-1.86±0.24	down
Csa2G264590	2 DBA	-2.41	-1.65±0.17	down
Csa7G378520	2 DBA	2.44	1.02±0.05	up
Csa2G081190	2 DBA	2.30	3.62±0.09	up
Csa3G431430	2 DBA	2.17	0.87±0.08	up
Csa6G147590	2 DBA	3.92	0.85±0.20	up
Csa4G556180	2 DBA	-2.25	-1.68±0.40	down
Csa5G374730	9 DAA	3.60	1.52±0.25	up
Csa2G381840	9 DAA	-5.82	-4.37±0.23	down
Csa4G556180	9 DAA	-2.58	-2.45±0.14	down
Csa1G572420	6 DBA	-2.09	-1.38±0.15	down
Csa1G572420	2 DBA	-1.84	-1.69±0.17	down
Csa1G572420	9 DAA	-7.00	-3.41±0.24	down
Csa3G011860	9 DAA	2.74	1.49±0.20	up
Csa3G012860	9 DAA	2.74	1.26±0.11	up
Csa3G903540	9 DAA	-9.50	-1.54±0.05	down
Csa3G179110	9 DAA	3.07	1.06±0.12	up
Csa3G535100	9 DAA	7.55	1.23±0.10	up
Csa7G391240	9 DAA	-3.87	-3.55±0.24	down
Csa3G116730	6 DBA	3.09	1.77±0.19	up
Csa5G155570	6 DBA	2.83	1.39±0.05	up
Csa2G349090	2 DBA	-1.96	-0.87±0.31	down
Csa1G340430	9 DAA	-2.56	-2.04±0.46	down
Csa5G167110	9 DAA	-2.95	-1.97±0.11	down
Csa1G039900	6 DBA	4.20	2.36±0.30	up
Csa1G051580	6 DBA	5.06	3.40±0.24	up
Csa4G358770	6 DBA	4.93	2.48±0.29	up
Csa6G076710	6 DBA	3.35	1.50±0.04	up
Csa1G039900	2 DBA	3.44	0.82±0.26	up
Csa1G051580	2 DBA	2.77	2.44±0.27	up
Csa3G179130	2 DBA	9.23	0.76±0.15	up
Csa6G076710	2 DBA	1.91	0.62±0.14	up
Csa1G524660	6 DBA	-2.16	-1.92±0.26	down
Csa1G009700	2 DBA	-2.61	-1.96±0.06	down
Csa2G352950	2 DBA	-2.10	-1.76±0.05	down
Csa5G152790	9 DAA	7.78	4.63±0.44	up
Csa3G199590	9 DAA	-3.18	-1.93±0.11	down
Csa5G515060	6 DBA	3.40	3.16±0.10	up
Csa1G033250	6 DBA	-2.29	-1.82±0.11	down
Csa3G859620	2 DBA	3.50	3.48±0.26	up
Csa1G033250	2 DBA	-2.56	-1.60±0.09	down
Csa2G352420	9 DAA	-4.36	-2.29±0.38	down
Csa7G049250	9 DAA	-2.73	-2.51±0.16	down

Supplementary Table 4: The content of IAA, zeatin and GA_3 in the fruits of *sf1* and WT

Sampling time	Samples	IAA content ($ng.g^{-1}$ DW)	Zeatin content ($ng.g^{-1}$ DW)	GA_3 content ($ng.g^{-1}$ DW)
6 DBA	WT	51.90±1.11a	89.52±0.00A	/
	sf1	46.94±2.06b	57.80±1.24B	/
2 DBA	WT	71.19±1.47A	86.88±0.79A	/
	sf1	60.78±1.69B	72.75±2.40B	/
9 DAA	WT	184.84±1.57B	2.50±0.09	88.79±1.71A
	sf1	197.99±1.25A	2.55±0.02	81.78±1.57B

Note: 'A/B' and 'a/b' indicate significant difference from WT at 0.01 and 0.05 probability levels, respectively (n=3).

Supplementary Table 5: The expression of genes in phenylpropanoid and carotenoid biosynthetic pathways of *sf1* vs. WT fruits by qPCR

Gene ID	Sampling time	log_2(mutant/ WT) from RNA-seq	log_2(mutant/WT) from qPCR	Gene expression up- or down-regulated	IPR annotation
Csa2G226860	9 DAA	7.41	2.92±0.43	up	β-Carotene-isomerase
Csa5G152820	9 DAA	2.82	2.62±0.02	up	CrtZ/R-b
Csa6G106700	9 DAA	-3.87	-0.44±0.06	down	CCD7
Csa4G064690	9 DAA	-3.01	-2.51±0.09	down	NCED
Csa1G435760	9 DAA	9.23	3.59±0.38	up	NCED
Csa2G433350	9 DAA	2.70	1.14±0.10	up	4CL
Csa4G091880	9 DAA	2.47	1.20±0.16	up	COMT
Csa4G045010	9 DAA	4.55	1.37±0.17	up	Peroxidase
Csa6G507230	9 DAA	3.51	1.97±0.23	up	Peroxidase
Csa7G058560	9 DAA	2.76	0.71±0.25	up	Peroxidase
Csa3G402970	9 DAA	-3.02	-0.84±0.14	down	BGLU
Csa1G372010	9 DAA	-2.67	-0.95±0.16	down	REF1
Csa6G501940	9 DAA	-7.32	-1.50±0.11	down	UGT72E

Supplementary Table 6: Primers used in map-based clone for *sf1* gene.

Chr.	Marker	Location	Forward primer	Reverse primer
Chr6	SSR11858	866,498-866,608	CCCTTCTCTCTCCTTCAATCC	GTTTGCATGGTGAAATGTGG
Chr6	SSR19672	4,943,628-4,943,784	AAGGCAGCAGAAAACTTGGA	CCCTCACTCTCGCTCACTCT
Chr6	SSR16451	9,628,895-9,629,047	CGTGGCATAAAACCACGAAT	TTTCATCAAATTCAACAAAACCC
Chr6	SSR22801	14,084,714-14,084,860	GGGTGAGACATAGTTCTGTGTGAA	CTTGACCAAGAGGTCAAAGC
Chr6	SSR16882	27,432,455-27,432,591	CACCTCAACTCCTCCATTCAA	TGGAGGTCATTGAGACTTGCT
Chr6	SSR21886	12,092,971-12,093,103	TCAGAGAAATGGAGAGGGAAA	CAGGATTTTTGTTTGGGGAA
Chr6	STS1	11,539,847-11,541,823	TGTTTGGCCCAAGGAGTT	GTAACTTGCATGCGAGGT
Chr6	STS2	11,597,234-11,600,926	AGAAGGGCTTGTGGGTGC	GAGACGATGAAGGGACAG
Chr6	STS3	11,608,600-11,609,761	TGGGATGGTGATCGAAAG	ATGAAGACGCCCTTACCG

Supplementary Table 7: Primers used in qPCR.

Gene ID	Forward primer	Reverse primer	IPR annotation
Csa2G264590	ATTGCCTGTCGTCATTCC	CACCAAAGCCAAACCCTA	AUX1
Csa5G374730	ATTTGGTCCTTCCTTGGC	AGCCCAGTAAACAGCAGAAG	AUX1
Csa7G378520	GTCGCCGATTCCAACCCT	AACATGGCAGCAAGAGCA	Aux/IAA
Csa2G381840	TGCCTTTGCTTTCTTGGA	GTTTGCGGAGGTTGAGTG	Aux/IAA
Csa4G556180	GCTGTGGAAGTAGGGTTG	ATCGGAGTTGGAGAAAGT	SAUR
Csa6G147590	ATGTGACCATAGCCAAGAA	CAAGCAACTCAACGGAAC	SAUR
Csa2G081190	ATGCTGGTGGTGTACTGA	TCTCCAATGGGTTGATAG	GH3
Csa3G431430	AATGCCAACAATAGAAGAAG	CGAGTTTAGAGTCCCAGTTC	GH3
Csa1G572420	AACCCAATTCCTTCGCTAC	TGCACTTTGTTTGCTCCA	HP
Csa3G903540	CCGTACACCTTTATTCAC	CTACTCCATTCCTCCAAC	KAO
Csa3G011860	TCTTTGGGATGAGGAAAT	GGCCATGAAACTACAGCT	KAO
Csa3G012860	GGTATTGATTACTGGAGGCT	CCAGGAGGAAGTTTAGGC	KAO
Csa3G179110	CTTATCCGTTCCTTATGTT	TCCACTATCTTCAAACCC	GA20ox
Csa3G535100	TGCTCGACCTTCCTCTGT	ATGCTCGCTAAAGCCAAT	GA2ox
Csa7G391240	GGCTGGGAGTAATGAAGT	ATACTCGGCTAACTCACG	GID1
Csa5G155570	GAATGAATGTAATGTGGGTAG	AAGATGGAGGAGATAGCAGT	AP2/ERF
Csa3G116730	GTCGTCGGATTTATTTCA	CTCCTCCTCACTCCTCTG	AP2/ERF
Csa2G349090	TTCCTCCAAACCCTCTTA	GAGATTGGCGTTCTTGTT	AP2/ERF
Csa5G167110	TTGAACCTCATCCGCTAC	GTGGGTCTTTGGACTTTG	AP2/ERF
Csa1G340430	TGCTTCCGATCATCAGTC	AAGGCAGTTCCTCTACCATT	AP2/ERF
Csa4G358770	AATCTGGGAAGAGGCTGTG	TGGCTGAATAGGCTGAAC	MADS
Csa6G076710	AGAATTGGTTCCAGGAGA	TCGATAGGGTCAGGTTGT	MADS
Csa3G179130	GAGGTATCACCAGCAATG	TTACTCCCACCGTCTATC	MADS
Csa1G051580	GAGGATCTCGAACCGTTGA	GCAGAGGCAGGATTAGCA	MADS
Csa1G039900	TTAGGTCCACTGAATGCC	TTGCGTTGAAGTTCTGTAAG	MADS
Csa1G524660	ACGCCGACGATCTCACTC	CCGTTGCCTCATCTCCTT	MYB
Csa1G009700	GGGAAGACCACCTTGTTG	CCCATTGACCCTTTGAGA	MYB
Csa2G352950	CTCCCATTGGCTCCTCAC	CTCCACCATTTGGCTGTT	MYB

Csa3G199590	CTGCCGTTTACGCTGGTT	TCGCATTGTCGGTTCGTC	MYB
Csa5G152790	CTCATCCGCTACATAACC	TTTCGTTGTCCGTTCGTC	MYB
Csa2G352420	AATGCTCCTCAATCCCACC	TGAGCGAAGAAACAGACCC	Zinc finger protein
Csa3G859620	TCTATGAGCGGCCAACTA	AAGAGTAAGGAGCCCAAT	Zinc finger protein
Csa5G515060	CGAAGAGGCAACAATCAT	CAAAGAAGGTGGCCGTAG	Zinc finger protein
Csa7G049250	GGTTGTGGTCGTGGTGAT	GCGTAGAAGTCGGTGAGG	Zinc finger protein
Csa1G033250	TGGATTCTAAAGACCCTAC	GGCGTCGTAGTAGTTTCT	Zinc finger protein
Csa2G226860	GCATTTCCAAAGCCAATC	TCGCAGGGTCCAACTAAC	β-Carotene-isomerase
Csa5G152820	CCGAGGGTTGGAGCATTT	AACGACGGTGAACGAGGC	CrtZ/R-b
Csa6G106700	ATGCCTCTTCTTGCTCAT	AACGAATCTCCGATTACC	CCD7
Csa4G064690	TGGTGAACCGAAATCTACTTG	CGAAGGCTAAGATGTGGC	NCED
Csa1G435760	ATGATTCACGCTGTCACTAT	CTTGGTAAGGAAGGTCGT	NCED
Csa2G433350	CGTTGCTCTGCCGTTCTC	TTGCCACCGTCACCTTGT	4CL
Csa4G091880	TGTCAGCGTTCCAAAGGG	GGGTTGTGAGCCAGCATT	COMT
Csa4G045010	GCTTAGGCTTCATTTCCA	AGCTTGGTCCTCCAGTAA	Peroxidase
Csa6G507230	CCTGGTGCCGACTTCATC	GACCTCCAGAAAGCACAACT	Peroxidase
Csa7G058560	TTTGTAGAGGGTTGTGATG	AACCAAGTCCTTTTCATTC	Peroxidase
Csa3G402970	ATTTTCCAGATGGTTTCA	ACAGTCTTGTTGGCATTG	BGLU
Csa1G372010	ATTCGTTGATTCCGTTTC	GCTGCCACTTCCTCCTTG	REF1
Csa6G501940	CTCCTCCGCAGAATCCAC	GAAGGGTAAAGATACGAGACAG	UGT72E

Supplementary Figure Legends

Supplementary Figure 1: Plant height (A), stem diameter at the fourth node from the plant base (B), leaf area of the first and the second functional leaves counting from the plant apex (C) and the first flower node (D) in WT and *sf1*. Plant height was defined as the distance from the plant base to the top bud. All parameters were measured at the flowering stage with 18 biological repeats. Vertical bars represent standard deviation.

Supplementary Figure 2: Influence of the *sf1* gene on cucumber fruit parameters. Spine height (A), height of spine base (B) and spine upper portion (C), and cell number of spine upper portion (D) in WT and *sf1* fruits at 11 DAA. Seed cavity (E) and ratio of seed cavity to fruit diameter (F) in WT and *sf1* fruits at 23 DAA. Stigma diameter (G) in WT and *sf1* fruits at 0 DAA. The contents of chlorophyll (H) and carotenoids (I) in WT and *sf1* at 11 DAA. '**'and '*' indicate significant differences from WT at the 0.01 and 0.05 probability levels, respectively. Vertical bars represent standard deviation (n = 3-12).

Supplementary Figure 3: Sarcocarp cell size of WT (A-C) and *sf1* (D-F) fruits at 0 (A and D), 9 (B and E) and 23 DAA (C and F). Scale bars represent 100 μm.

Supplementary Figure 4: The expression of annotated genes in the fine-mapped region on chromosome 6 in WT and *sf1* fruits. Total RNA was extracted from WT and *sf1* fruits at 6 DBA, 2 DBA and 9 DAA. The expression of 12 annotated genes (*Csa6G176930*, *Csa6G176940*, *Csa6G177440*, *Csa6G178440*, *Csa6G178940*, *Csa6G178950*, *Csa6G179450*, *Csa6G179460*, *Csa6G179470*, *Csa6G179480*, *Csa6G181000*, and *Csa6G181510*) was detected by semi-quantitative RT-PCR. The semi-quantitative RT-PCR was performed in triplicate with a housekeeping gene, *actin*, in parallel as the internal control.

Supplementary Figure 5: Venn diagrams showing genes that were differentially expressed in the cucumber fruits of mutant vs. WT groups at 6 DBA, 2 DBA and 9 DAA.

Supplementary Figure 6: DEG-enriched phenylpropanoid biosynthetic pathway from KEGG analysis (p-value < 0.01) in fruit samples of mutant vs. WT group at 9 DAA. The red closed rectangles represent up-regulated genes, and the green closed rectangles represent down-regulated genes. The number of rectangles indicates the fold gene expression difference in fruits of the mutant group compared to the WT group. PAL, phenylalanine ammonia-lyase; BGLU, β-glucosidase; C4H, cinnamate 4-hydroxylase; C3H, coumarate 3-hydroxylase; COMT, catechol O-methyltransferase; 4CL, 4-coumarate:coenzyme A ligase; F5H, ferulate 5-hydroxylase ; REF1, reduced epidermal fluorescence 1; CCR, cinnamoyl-CoA reductase; CAD, cinnamyl alcohol dehydrogenase.

Supplementary Figure 7: DEG-enriched carotenoid biosynthetic pathway from KEGG analysis (p-value < 0.01) in fruit samples of mutant vs. WT group at 9 DAA. The red closed rectangles represent up-regulated genes, and the green closed rectangles represent down-regulated genes. The number of rectangles indicates the fold gene expression difference in fruits of the mutant group compared to the WT group. PSY, phytoene synthase; NCED, 9-cis-epoxycarotenoid dioxygenase; ABA, abscisic acid.

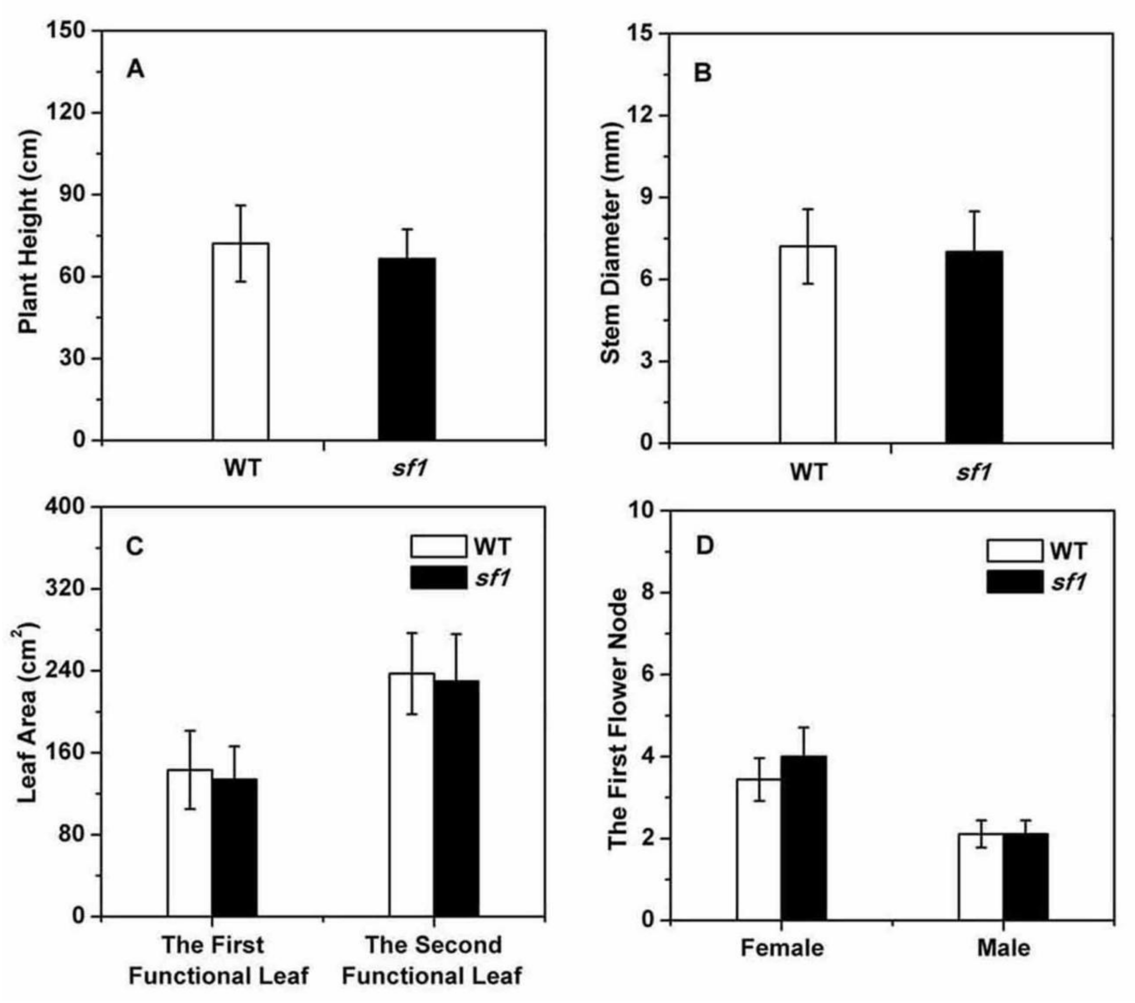
A
Plant Height (cm)
150
120
90
60
30
0
WT
sf1
B
Stem Diameter (mm)
15
12
9
6
3
0
WT
sf1
C
Leaf Area (cm2)
400
320
240
160
80
0
WT
sf1
The First Functional Leaf
The Second Functional Leaf
D
The First Flower Node
10
8
6
4
2
0
WT
sf1
Female
Male

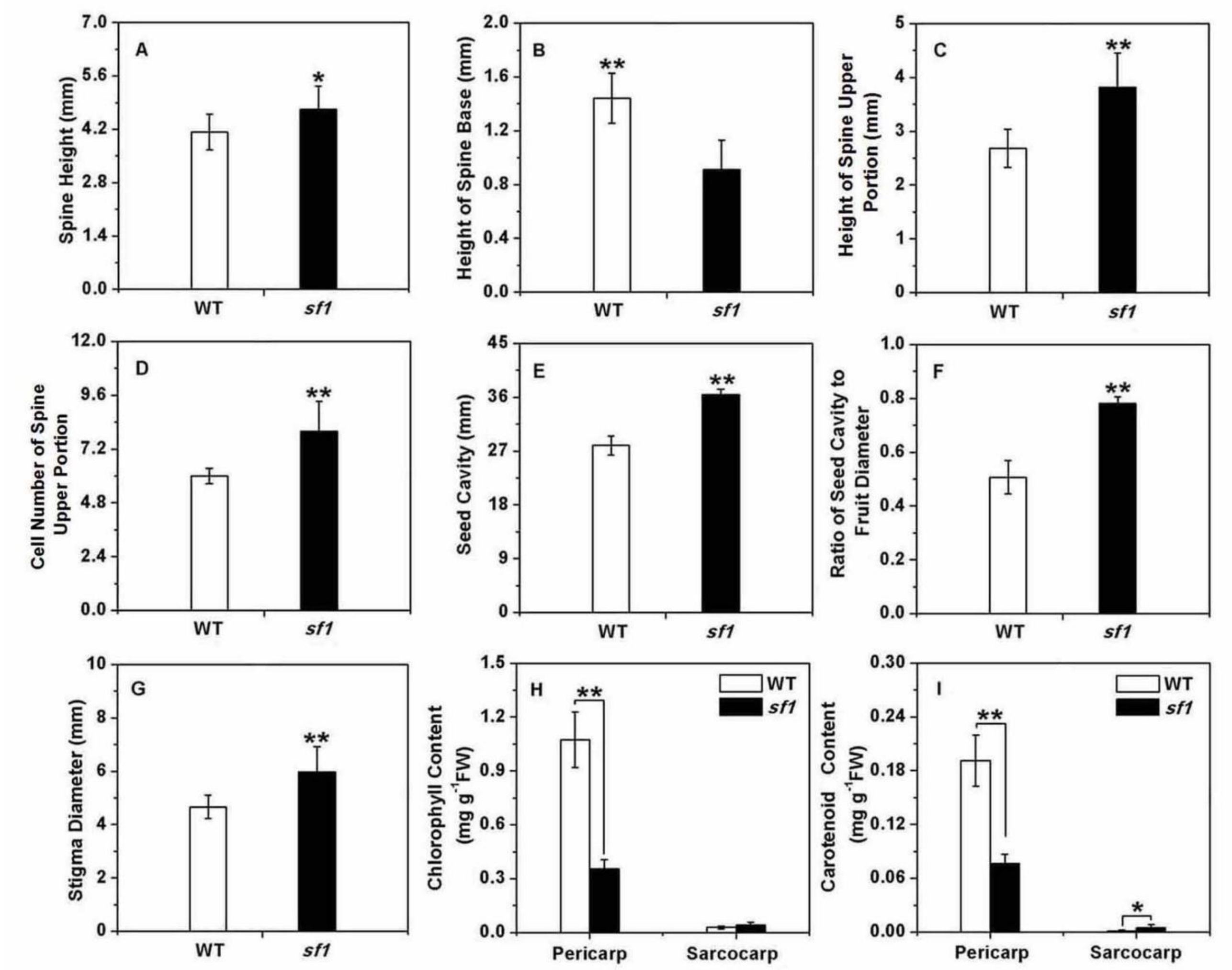
A
Spine Height (mm)
WT
sf1
*
B
Height of Spine Base (mm)
**
C
Height of Spine Upper Portion (mm)
**
D
Cell Number of Spine Upper Portion
**
E
Seed Cavity (mm)
**
F
Ratio of Seed Cavity to Fruit Diameter
**
G
Stigma Diameter (mm)
**
H
Chlorophyll Content (mg g-1FW)
Pericarp
Sarcocarp
I
Carotenoid Content (mg g-1FW)
*

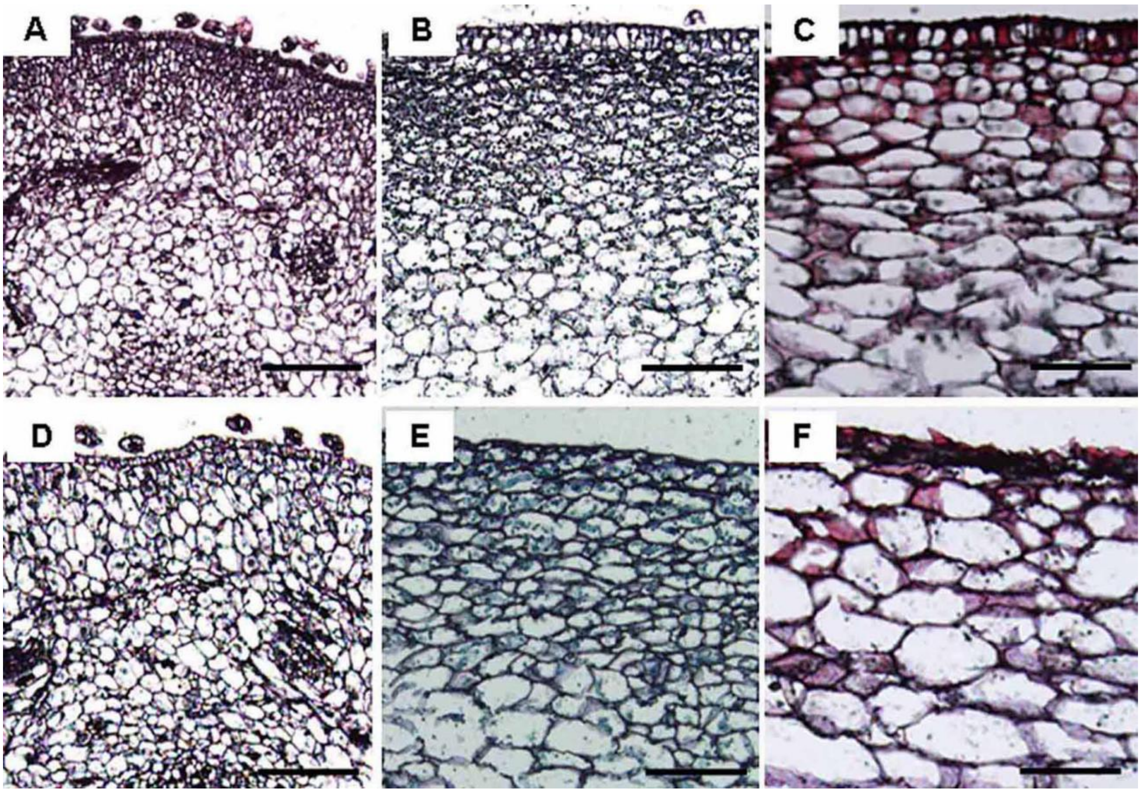
A
B
C
D
E
F

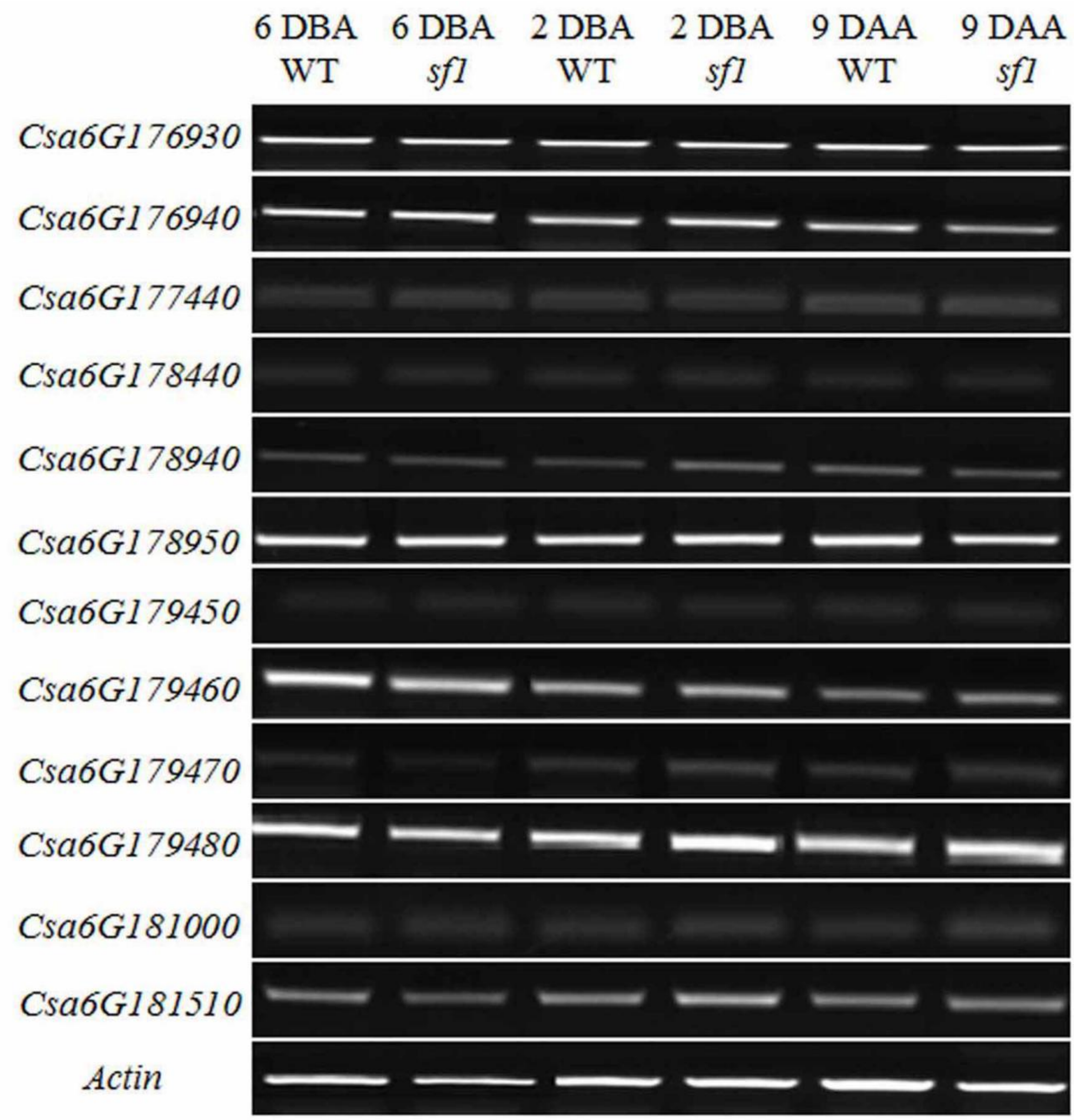
6 DBA WT
6 DBA sf1
2 DBA WT
2 DBA sf1
9 DAA WT
9 DAA sf1
Csa6G176930
Csa6G176940
Csa6G177440
Csa6G178440
Csa6G178940
Csa6G178950
Csa6G179450
Csa6G179460
Csa6G179470
Csa6G179480
Csa6G181000
Csa6G181510
Actin

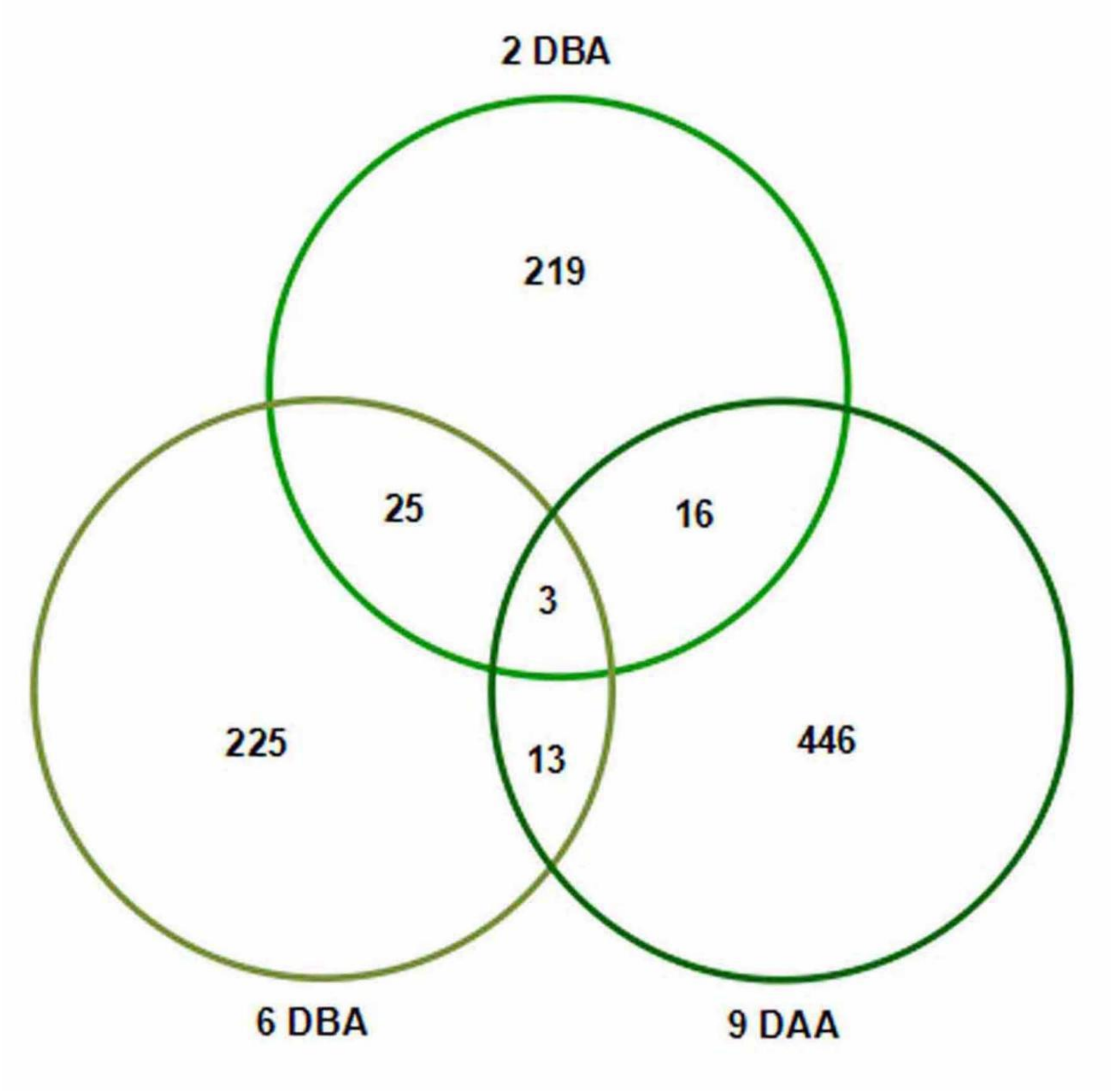
2 DBA
219
25
16
3
225
13
446
6 DBA
9 DAA

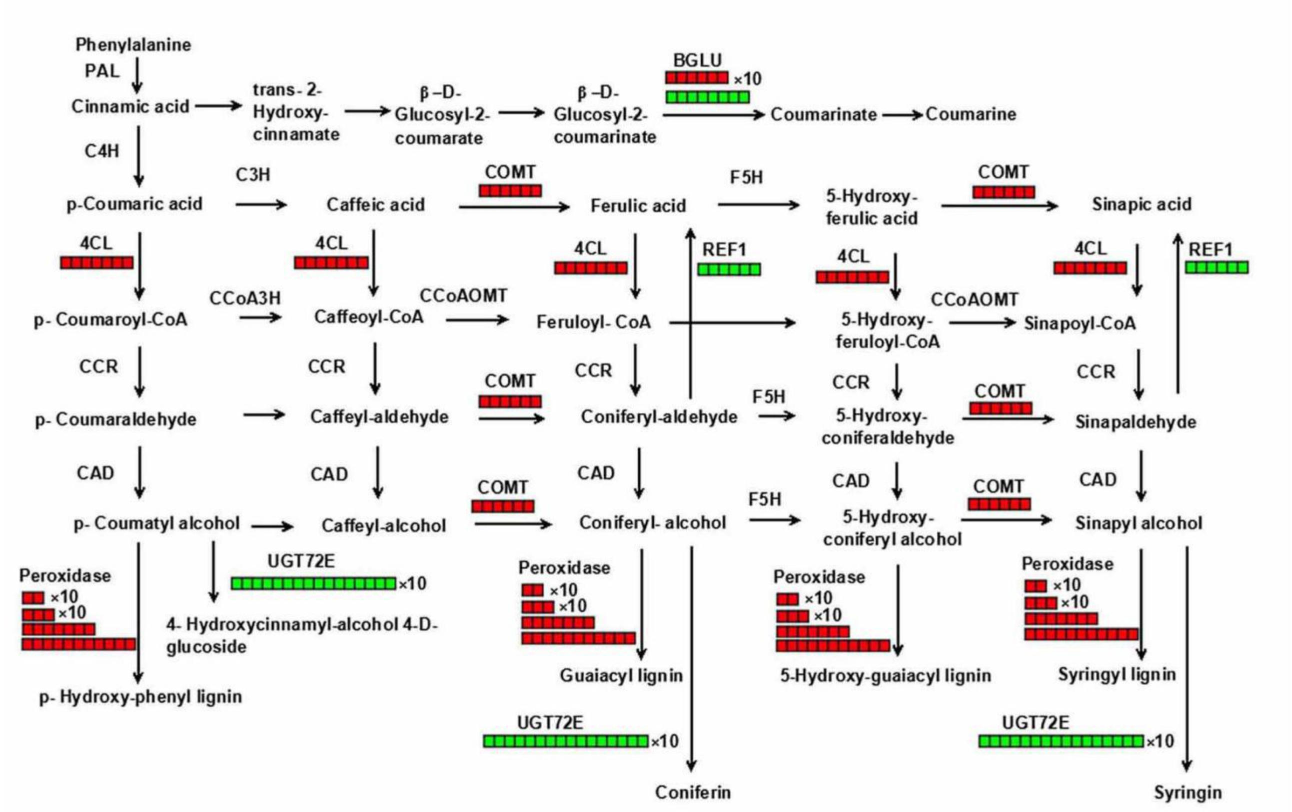

Phenylalanine
PAL
Cinnamic acid
trans- 2-Hydroxy-cinnamate
β –D-Glucosyl-2-coumarate
β –D-Glucosyl-2-coumarinate
BGLU
×10
Coumarinate
Coumarine
C4H
p-Coumaric acid
C3H
Caffeic acid
COMT
Ferulic acid
F5H
5-Hydroxy-ferulic acid
COMT
Sinapic acid
4CL
4CL
4CL
REF1
4CL
4CL
REF1
p- Coumaroyl-CoA
CCoA3H
Caffeoyl-CoA
CCoAOMT
Feruloyl- CoA
5-Hydroxy-feruloyl-CoA
CCoAOMT
Sinapoyl-CoA
CCR
CCR
COMT
CCR
F5H
CCR
COMT
CCR
p- Coumaraldehyde
Caffeyl-aldehyde
Coniferyl-aldehyde
5-Hydroxy-coniferaldehyde
Sinapaldehyde
CAD
CAD
COMT
CAD
F5H
CAD
COMT
CAD
p- Coumatyl alcohol
Caffeyl-alcohol
Coniferyl- alcohol
5-Hydroxy-coniferyl alcohol
Sinapyl alcohol
Peroxidase
×10
×10
UGT72E
×10
4- Hydroxycinnamyl-alcohol 4-D-glucoside
Peroxidase
×10
×10
Peroxidase
×10
×10
Peroxidase
×10
×10
p- Hydroxy-phenyl lignin
Guaiacyl lignin
5-Hydroxy-guaiacyl lignin
Syringyl lignin
UGT72E
×10
UGT72E
×10
Coniferin
Syringin

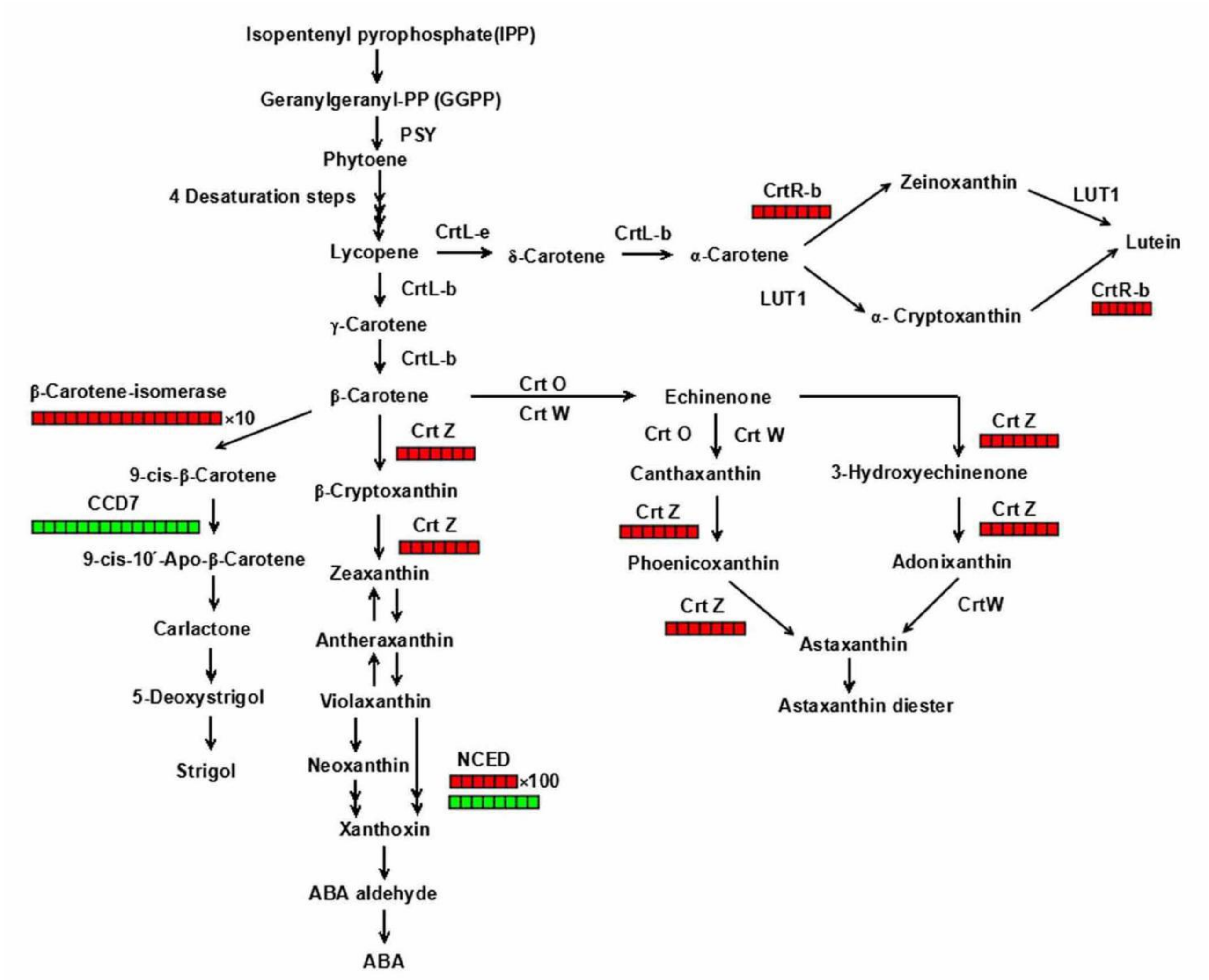
Isopentenyl pyrophosphate(IPP)
Geranylgeranyl-PP (GGPP)
PSY
Phytoene
4 Desaturation steps
Lycopene
CrtL-e
δ-Carotene
CrtL-b
α-Carotene
CrtR-b
Zeinoxanthin
LUT1
Lutein
LUT1
α- Cryptoxanthin
CrtR-b
CrtL-b
γ-Carotene
CrtL-b
β-Carotene-isomerase
×10
β-Carotene
Crt O
Crt W
Echinenone
Crt Z
Crt O
Crt W
Crt Z
9-cis-β-Carotene
β-Cryptoxanthin
Canthaxanthin
3-Hydroxyechinenone
CCD7
Crt Z
Crt Z
Crt Z
9-cis-10´-Apo-β-Carotene
Zeaxanthin
Phoenicoxanthin
Adonixanthin
Crt Z
CrtW
Carlactone
Antheraxanthin
Astaxanthin
5-Deoxystrigol
Violaxanthin
Astaxanthin diester
Strigol
Neoxanthin
NCED
×100
Xanthoxin
ABA aldehyde
ABA
